# Fungal drops: a novel approach for macro- and microscopic analyses of fungal mycelial growth

**DOI:** 10.1093/femsml/uqad042

**Published:** 2023-10-18

**Authors:** Matteo Buffi, Guillaume Cailleau, Thierry Kuhn, Xiang-Yi Li Richter, Claire E Stanley, Lukas Y Wick, Patrick S Chain, Saskia Bindschedler, Pilar Junier

**Affiliations:** Laboratory of Microbiology, University of Neuchâtel, Rue Emile-Argand 11, 2000 Neuchâtel, Switzerland; Laboratory of Microbiology, University of Neuchâtel, Rue Emile-Argand 11, 2000 Neuchâtel, Switzerland; Laboratory of Microbiology, University of Neuchâtel, Rue Emile-Argand 11, 2000 Neuchâtel, Switzerland; Laboratory of Eco-Ethology, University of Neuchâtel, Rue Emile-Argand 11, 2000 Neuchâtel, Switzerland; Laboratory of Microbiology, University of Neuchâtel, Rue Emile-Argand 11, 2000 Neuchâtel, Switzerland; Laboratory of Eco-Ethology, University of Neuchâtel, Rue Emile-Argand 11, 2000 Neuchâtel, Switzerland; Department of Bioengineering, Imperial College London, B304, Bessemer Building, South Kensington Campus, SW7 2AZ, London, United Kingdom; Department of Environmental Microbiology, Helmholtz Centre for Environmental Research - UFZ, Permoserstrasse 15, 04318 Leipzig, Germany; Bioscience Division, Los Alamos National Laboratory, Los Alamos, P.O. Box 1663, NM 87545, United States; Laboratory of Microbiology, University of Neuchâtel, Rue Emile-Argand 11, 2000 Neuchâtel, Switzerland; Laboratory of Microbiology, University of Neuchâtel, Rue Emile-Argand 11, 2000 Neuchâtel, Switzerland

**Keywords:** bacterial–fungal interactions, mycelium observation, quantification fractal dimension, fungal highways, mycological method, user-friendly

## Abstract

This study presents an inexpensive approach for the macro- and microscopic observation of fungal mycelial growth. The ‘fungal drops’ method allows to investigate the development of a mycelial network in filamentous microorganisms at the colony and hyphal scales. A heterogeneous environment is created by depositing 15–20 µl drops on a hydrophobic surface at a fixed distance. This system is akin to a two-dimensional (2D) soil-like structure in which aqueous-pockets are intermixed with air-filled pores. The fungus (spores or mycelia) is inoculated into one of the drops, from which hyphal growth and exploration take place. Hyphal structures are assessed at different scales using stereoscopic and microscopic imaging. The former allows to evaluate the local response of regions within the colony (modular behaviour), while the latter can be used for fractal dimension analyses to describe the hyphal network architecture. The method was tested with several species to underpin the transferability to multiple species. In addition, two sets of experiments were carried out to demonstrate its use in fungal biology. First, mycelial reorganization of *Fusarium oxysporum* was assessed as a response to patches containing different nutrient concentrations. Second, the effect of interactions with the soil bacterium *Pseudomonas putida* on habitat colonization by the same fungus was assessed. This method appeared as fast and accessible, allowed for a high level of replication, and complements more complex experimental platforms. Coupled with image analysis, the fungal drops method provides new insights into the study of fungal modularity both macroscopically and at a single-hypha level.

## Teaser

Development of an inexpensive and fast method to study and quantify modular growth in filamentous microorganisms at both the mycelial and hyphal scales.

## Introduction

Filamentous fungi are tip-growing organisms that display a three-dimensional (3D) radial growth of tube-like branched structures called hyphae (Grove and Bracker 1970). This growth pattern eventually leads to the formation of mycelial networks, which are structures observed from the millimetric to the metric-scale. This growth mechanism is ideal for the colonization of soils, and thus, it is observed in other fungi-like microorganisms including some bacteria and protists (Wolf et al. 2013, Geisen et al. 2018). Soils are considered as one of the most structurally and biologically complex ecosystems on Earth and a major reservoir of biodiversity (Nannipieri et al. 2003, Fitter et al. 2005, Senesi and Wilkinson 2008, Phillips 2017). In soils, shifting chemical properties and heterogeneity are exacerbated by an ever-changing physical matrix, which gives rise to a 3D structure with an arguably infinite combination of microniches (Ettema and Wardle 2002, Tiedje et al. 2009). Given the complexity of soils, fungi and fungi-like organisms need to be able to coordinate the behaviour of their mycelial network in order to cope with all the interactions and challenges encountered during colonization. Filamentous fungi have a remarkable ability to adapt to the variable 3D structure of soils. This is provided by the secondary growth of hyphae, called branching, and by the process of hyphal anastomosis, consisting in cell-to-cell fusion at the hyphal scale. In addition to this, filamentous fungi are also able to reallocate resources to regions of the mycelia where nutrients are required, leaving some hyphae almost devoid of cytoplasmic content. Eventually, some parts of the mycelial network may even be used as a nutrient source through autolysis (Jiang et al. 2022). All these processes allow fungi to reorganize their network in order to improve resource exploitation through nutrient acquisition and translocation (Rayner et al. 1995, Harris 2008, Fischer and Glass 2019). Network reorganization through branching, anastomosis, and autolysis are thus essential process that enable fungal survival and colonization of environments with a complex 3D structure (Fricker et al. 2007, Harris 2008).

To understand the process of network reorganization, the 3D structure and complexity of soils must be replicated. However, achieving this at a laboratory scale is highly challenging. Solid media is generally used for the growth and maintenance of fungal mycelia. Solid media can be modified to allow for the combination of multiple nutrient conditions or the separation of interacting organisms, for example in two-compartment Petri dishes (Hunziker et al. 2015). However, in solidified media (e.g. agar-based media), direct microscopical observation of individual hyphae is difficult due to extensive and overlapping mycelial growth. Cutting of a thin media layer and observation on a glass slide allows for the recognition of physiological structures such as sexual or asexual fructifications. But the spatial arrangement of the sample cannot be preserved during preparation of thin slices. Furthermore, nutrient heterogeneity, which is a defining feature of soils (Aleklett et al. 2018), is difficult to replicate in jellified media. Cultivation in liquid media is often used for the production of metabolites by fungi, but liquid media is an even poorer representation of soils. In fully mixed liquid cultures, emulating the patchiness of nutrient distribution is not possible and microscopic observations are also difficult. The use of microfluidic devices allows for spatial separation and confinement of single hyphae under nutrient-rich and deficient conditions, but provides only limited control of air-filled voids (Gimeno et al. 2021). Furthermore, microfluidic technologies often require specific equipment for their production. Other techniques are being developed to recreate soil in a 2D or 3D fashion (Otten et al. 2012, Aleklett et al. 2018), but experimental systems that allow to replicate more than one or two soil properties simultaneously are uncommon.

In this study, a novel and inexpensive approach for the macro- and microscopic observation of fungal mycelia is proposed. The approach aims to recreate the patchy structure of soil in a simple and straightforward manner. This provides a way to replicate niche heterogeneity associated with air gaps and assorted resource availability. By disposing drops of liquid media on a cell culture-treated Petri dish, it is possible to recreate a 2D patchy environment for the fungus to grow. The individual drops, each of a few microliters, allow for multistimuli testing on one mycelial colony at once. The control of distance between the drops allows for the observation of mycelial exploration at the colony (macroscopic) or single hyphal (microscopic) levels. Inoculation can be done either using spores or mycelial fragments, recreating a situation where a single propagule starts to explore its environment. Also, the system can be used for the characterization of different mycelial behaviours, such as branching, elongation-growth speed, surface hyphal proprieties (i.e. hydrophilicity or hydrophobicity), thigmotropism, formation of reproductive structures, or secondary growth. As the drops are not confined, the addition of other factors (organisms, stressors, and nutrients) is possible at any time during the experiment. This is particularly suited for observing response to an abiotic or trophic factor, and intraorganismal interactions. Furthermore, as continuous microscopic observations are feasible, it is possible to produce time-lapse images and evaluate the complexity of the mycelial network over time by quantifying the changes in the mycelial mass fractal dimension (FD). FD is an index that represents in a single value the complexity and space-filling efficiency of mycelial growth in response to different stimuli at a microscopic scale (Juge et al. 2009). This method is already used to quantify shape in corals (Martin-Garin et al. 2007), complexity during plants development (Corbit and Garbary 1995), and space-filling capabilities in filamentous microorganisms (Boddy et al. 1999, Papagianni 2006, Barry et al. 2009). The latter is particularly relevant for filamentous fungi in which a trade-off between apical and lateral hyphal growth is essential for resource exploitation, exploration, and colonization intensity of highly heterogeneous substrates (Camenzind et al. 2020). Moreover, the development and use of this method to extract information on the network features of mycelial growth has greatly facilitated the quantitative description of the complex behaviour of filamentous microorganisms and our ability to characterize these dynamic networks (Heaton et al. 2012). FD analysis requires high consistency in image collection and treatment within a given experiment, and is especially suited for describing complex structures on a flat surface (Juge et al. 2009). All of the former makes FD suitable as a quantitative approach to analyse the results generated with the spore drop method, which typically will consist in images of filamentous network with different levels of complexity. Herein, the validation of the approach is described by testing three different scenarios: (i) growth and features’ observation for different filamentous fungal species and one oomycete, (ii) modulation of the mycelial network of a selected filamentous fungus to different nutrient concentrations, and (iii) bacterial–fungal interactions. The two latter scenarios were chosen to provide not only a proof-of-concept for the fungal drops method, but also to demonstrate its general application to obtain insights into the morphophysiological and ecological responses of fungi and other filamentous microbial species to changing trophic and biotic factors in a complex environment. Indeed, soil heterogeneity, both in terms of structure and resource distribution as well as bacterial–fungal interactions are known to be key to soil functioning and fungal ecology (Barron 1988, 2003, De Boer et al. 2005, Deveau et al. 2018). Hence, the scenarios chosen. Moreover, this new approach can be replicated with ease in any laboratory using basic microbiological equipment and without the need for specialized materials.

## Materials and methods

### Method validation

To initially test if the fungal drops method would function for different filamentous microbial species, five organisms were selected from our laboratory collection. Three Ascomycota fungi: the phenotypic plastic *Fusarium oxysporum*, the well-known gourmet mushroom *Morchella crassipes*, and the saprotrophic biocontrol agent *Trichoderma rossicum*; the fast growing and ubiquitous *Mucor moelleri* (Mucoromyctoa); and the pathogenic Oomycota *Pythium ultimum*. In all further experiments, *F. oxysporum* was used as main model organism. This fungus is commonly found in soils and its very large phenotypic plasticity makes it an interesting model to study fungal behaviour. Some strains are described as plant pathogens (Fravel et al. 2003, Gordon 2017, Joshi 2018), while others have been described as beneficial (or at least neutral) to plants (de Lamo and Takken 2020). Moreover, easy spore production was also an important factor in the choice of this organism. For the experiments testing bacterial–fungal interactions, the model soil bacterium *Pseudomonas putida* KT2440 (Nelson et al. 2002) was used. This bacterium has been used in previous experiments investigating interactions with soil fungi (Pion et al. 2013) or fungi-like soil dwellers (You et al. 2021). The strain used is constitutively tagged with green fluorescent protein (GFP). All the strains were obtained from the fungal and bacterial collection of the laboratory of microbiology, University of Neuchâtel, Switzerland. For the regular maintenance, all filamentous microorganism were plated on Malt Extract Agar (MEA; Table S1, Supporting Information), while the bacterium was maintained on Nutrient Agar (NA; Table S1, Supporting Information).

### Inocula preparation

For experiments involving *P. ultimum, M. moelleri*, and *M. crassipes*, mycelial suspensions were used for drop inoculation due to the difficulty in producing and/or collecting asexual spores. *P. ultimum* and *M. moelleri* were inoculated in M9 mineral liquid medium (Table S1, Supporting Information) while Malt Extract Broth (MEB; Table S1, Supporting Information) was used for *M. crassipes*. All fungi were then cultured at room temperature under agitation (Lab Shaker, Adolf Kühner AG) at 120 rpm for 5 days. The mycelium was then fragmented in a 50-ml Falcon tube (CORNING®) using an ULTRA-TURRAX® (IKA® T18 basic) at max speed for 10 seconds, and then washed three times with physiological water (0.9 g l^−1^ NaCl). Hyphal density was then assessed with a Neubauer chamber (BIOSYSTEMS® 0.01 mm) and resuspended at a final concentration of around 10 hyphal fragments l^−1^ in M9 mineral medium (Table S1, Supporting Information) for *P. ultimum* and *M. moelleri* and MEB for *M. crassipes*. For experiments involving *F. oxysporum* and *T. rossicum*, spore suspensions were used for drop inoculation. *Fusarium oxysporum* was grown on Potato Dextrose Agar (PDA; Table S1, Supporting Information) for 2 weeks in order to induce asexual spore formation. Spores were then collected following a method described previously (Bruisson et al. 2019) and stored in 500 µl MilliQ water at 4°C. Spores were quantified in a Neubauer Chamber (BIOSYSTEMS® 0.01 mm) and diluted to the desired concentration in MilliQ water or in the selected medium immediately before the start of the experiment. The same general method was used for *T. rossicum*, with the only difference being the induction of sporulation. For conidia production, *T. rossicum* was inoculated on MEA (Table S1, Supporting Information) and incubated at room temperature under the exposition to sunlight for 2 weeks in order to increase conidia production. The bacterium *P. putida* KT2440 was cultivated overnight in 5 ml of Nutrient Broth (NB; Table S1, Supporting Information), at 120 rpm room temperature. The culture was then washed three times with physiological water and diluted to ∼2.2 × 10^4^ cells in the target medium as a final inoculum.

### Construction of the system

Tissue-culture treated Petri dishes (CORNING®, REF 430167) compatible with direct inverted microscopic observations were used. These Petri dishes are treated for optimal cell adhesion, and are often used for the culturing of mammal cells. Masks to facilitate the precise positioning of the drops were made beforehand with Adobe illustrator® and are provided as Supplementary Information (Figure S1, Supporting Information). Deposition of the drops (15–20 µl) was performed with a 20-µl pipette and with the aid of the mask placed underneath the Petri dish. To prevent drop desiccation a larger Petri dish with humidified cotton or an incubation chamber was prepared using either a desiccator containing moistened vermiculite at the bottom. In the desiccator humidity was measured using a hygrometer (GFTH200, Greisinger®). Air circulation was maintained using a hydrophobic filter allowing for gas exchange while retaining moisture inside.

## Comparison of the fungal drops system to normal agar medium

To compare the growth and the utility of the fungal drops system to normal agar based solid media, spores of *F. oxysporum* were collected as mentioned above and suspended to 1–2 spores per 15 µl in liquid MEB (Table S1, Supporting Information). For this, 15 µl drops where then deposed either on solid PDA or on tissue culture-treated Petri dishes (CORNING®, REF 430167) and then incubated at room temperature. To compare the two systems, pictures were taken in the same time frame (8-day post inoculation or dpi). Additionally, a 24-hour time-lapse was performed between 1 and 2 dpi to show how the fungus escapes the drop environment and further colonizes the space between the drops.

### Stereo- and microscopic analysis of the system

Whole Petri dish images were taken with a Canon Powershot SX230 HS camera. Macroscopic observations of single drops were performed with a stereoscope (NIKON SMZ18). Microscopical observations were performed with an inverted microscope (EVOS FL, EVOS M5000, Invitrogen) at room temperature and room relative humidity. For this, the Petri dish was positioned on the microscope and the focus was performed manually.

### Measure of drop area for each media used at different volumes

In order to assess the reproducibility of the system, different drop volumes (5, 10, 15, and 20 µl) for each liquid medium used for this manuscript (Table S1, Supporting Information) were deposed in triplicates on a tissue culture-treated Petri dish (CORNING®, REF 430167), and then photographed with a Canon Powershot SX230 HS with the aid of a camera holder. The pictures were taken on a flat levelled surface to avoid the change in shape of the drop during visualization. The area in cm^2^ was then measured with Image J and the results were plotted with R version 4.2.2 (R Core Team 2022).

### Fluorescein trace test for assessing drop leakage on mycelium

To observe whether any hydraulic flow established from one drop to another trough fungal hyphae, *M. moelleri* was inoculated as mentioned above and 0.01% Fluoresceine sodium (MERCK Sigma-Aldrich, Germany, Ref: 518–47-8) was added either in the inoculum or the target drops. This molecule is often used as a fluorescent tracer for liquids. Images with a GFP filter were taken in order to follow the liquid movement. The same experiments were performed with *P. ultimum*.

### Effect of target nutrient availability on mycelial growth of *F. oxysporum*

#### System set-up

In order to investigate the behaviour of *F. oxysporum* when confronted with different nutrient concentrations, asexual spores of the fungus were collected and quantified as described above and then diluted in MEB (Table S1, Supporting Information) to a concentration of about two spores µl^−1^. The drop system was prepared using a flower-like design (mask provided in Figure S1, Supporting Information) in order to assess the effect of different nutrient concentrations on growth patterns. The central drop (15 µl inoculum) was surrounded by six drops (15 µl each) containing three different target media: potato dextrose broth (PDB; Table S1, Supporting Information), PDB diluted at a 1:2 ratio (PDB 1:2) or 1:10 ratio (PDB 1:10). Drops with the different media were all positioned 5 mm from the central drop and in duplicates for each flower. Positioning of the different target media were randomized three times in order to avoid experimental bias. Each Petri dish contained six identical flowers, for a total of six Petri dishes giving 36 replicates for each target media.

#### Image treatment and mass FD analysis

To assess mass FD of the mycelial network image treatment was performed using R version 4.2.2 (R Core Team 2022) and Image J (Schneider et al. 2012). FD was measured using the box counting method already used for mycelia (Obert et al. 1990, Boddy et al. 1999, Senesi and Wilkinson 2008). This method involves overlapping the image with grids of scaling pixel sizes (i.e. 3, 6, 12, 24, 48, 96, 192, and 384). Mycelial presence for each grid box and at a given size was then assessed and FD was estimated as the slope of the logarithmic regression between the counted boxes and their scaling factor. For the mass FD, the images needed to be treated to extract the mycelium from the image and to remove unwanted noise around the fungal filaments. Step-wise treatment was performed as follows: (i) 8-bit images were hand-cropped in R to extract the region of interest removing the scalebar and drops from the image to be analysed, (ii) a Kuwahara smoothing filter (linear variant) was selected in ImageJ to perform an adaptive noise reduction to highlight the hyphae, (iii) a Sobel edge detector was used to detect drastic changes in image intensity (i.e. the hyphae), (iv) the output was converted to a binary mask that makes the fungal filament edge appear white and the background black, and (v) for each grid made of a given box size, each box covering a white pixel was counted. After repeating step five over the range of box sizes, the mass FD of the image was calculated. All the scripts used are provided as a detailed procedure (Supplementary Information S1). Statistical analyses were performed in RStudio (RStudio 2020). A two factor ANOVA was performed, and a *post hoc* Tukey contrast was used to obtain pair-wise comparisons between the different factors for the exploratory behaviour based on nutrient choice.

#### Image treatment and colour quantification

To quantify the red colouration in relation to different media concentrations, the images were processed using ImageJ software to isolate the red colour from the rest of the image. First, the images were converted into LAB colour space using the ‘Lab stack’ function in ImageJ. The ‘a-chroma’ stack was then duplicated and extracted from the stacks. Next, an automatic thresholding algorithm called the Huang Thresholding Algorithm was applied to all images to maximize the entropy between the pixels that needed to be counted (i.e. red colour) and the background pixels. The Polygon selection tool in ImageJ was used to define the border of the drops as the area of interest for analysis. To quantify the red pixels, the ‘Analyse Particles’ function in ImageJ was used for each defined drop. Drops that were not fully visible in the image were excluded from the analysis. Statistical analyses were performed as indicated above for the FD analysis.

### Bacterial–fungal interactions

#### System set-up

In order to assess the interaction of *F. oxysporum* with the bacterium *P. putida*, asexual spores of the fungus and cells of *P. putida* KT2440 were prepared as described above and then resuspended in MEB to a final concentration of one spore µl^−1^ for *F. oxysporum* and 2 × 10^4^ bacterial cells per drop for *P. putida* KT2440. The drop system was prepared as described above using lanes of four consecutive drops with a 5-mm gap between each drop. Three lanes were placed per Petri dish, each placed 2 cm apart from the other (mask provided in Figure S1, Supporting Information). The experimental setting included three different conditions: (i) simultaneous inoculation of both the spores and the bacterial cells in the same drop, (ii) inoculum of *F. oxysporum* alone, and (iii) inoculum of *P. putida* alone. To control for the change in volume upon bacterial coinoculation, 10 µl of sterile physiological water (0.9% NaCl in deionized water) were added to the fungus-alone condition.

#### Bacterial viability

To verify the dispersal and viability of bacteria, bacterial abundance in the drops was measured each day for 8 days in three independent replicates. For this, 4 µl were collected and suspended in 16 µl of NB. The resulting suspension was then diluted six times by resuspending each time 10 µl in 90 µl of NB (10x dilution series). A volume of 5 µl of each dilution were then plated on NA enriched with cycloheximide (500 mg l^−1^) and incubated at 30°C. After 24 hours, bacterial abundance was assessed by counting colony forming units (CFU).

#### FD analysis

In order to quantify the effect of *P. putida* on the growth of *F . oxysporum* FD differences were measured as described above. Pictures from six replicates were taken in between the inoculum drop and the first target drop after 7 days for the conditions in which the fungus was inoculated alone and when coinoculated with the bacterium. A Student’s *T*-test is used to compare the mean between the two groups. All the statistical analyses and plotting are performed on RStudio (RStudio 2020).

## Results

### Construction of the fungal drops system

**Figure 1.**
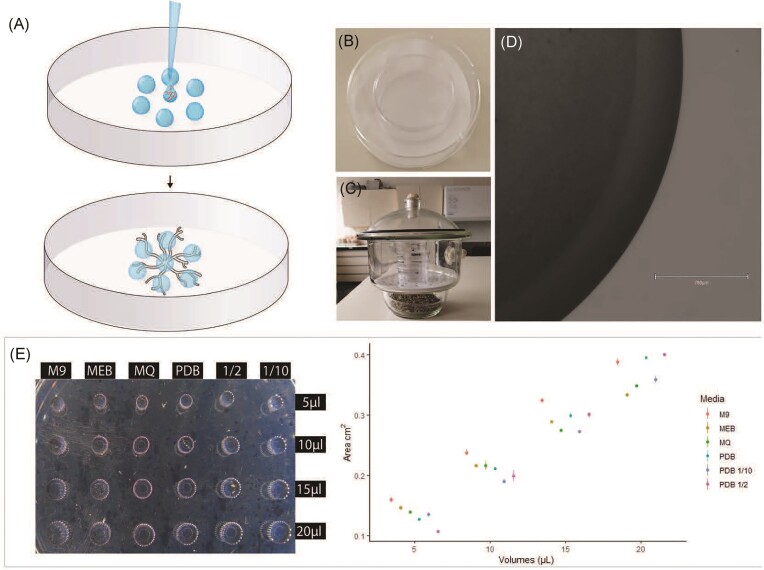
Experimental set-up of the drop system. (A) Schematic representation of the system: one or multiple drops consists of the spore or mycelial fragments’ inoculum (here drop in the middle), which is surrounded by other drops to be colonized and monitored. In order to maintain humidity two different humidity-controlled chambers were used: (B) a 9-mm Petri dish with a cotton filter soaked in sterile water; or (C) a desiccator containing water-saturated vermiculite at the bottom and a hydrophobic air filter. (D) Microscopical image of noninoculated drop. In the image it is possible to observe the continuous border of the drop, showing no diffusion of the liquid on the treated surface. (E) Image showing drops of different volume (5, 10, 15, and 20 µl) of all the different media used in this work deposed on the cell culture-treated Petri dish (left); a plot relating the area (cm^2^) measured for each different volume (three replicates for each volume) is shown on the right. Each coloured point represents the mean and standard deviation for three replicates of the same media, the different colours represent different media tested. Red: M9 mineral medium (M9); yellow: MEB; green: Milli Q water (MQ); light blue: PDB; dark blue: PDB 10 times diluted (PDB 1/10); and violet: PDB two times diluted (PDB 1/2).

The goal of the approach developed here was to recreate the heterogeneous structure and patchy resource distribution of soils in order to observe the exploratory behaviour and growth of filamentous microorganisms. For this, a variable number of drops were deposited at a fixed distance on a cell culture Petri dish treated to improve cell adhesion. The premise considered here is that given the radial and exploratory growth pattern of filamentous fungi, they will exit the initial drop and colonize adjacent drops. Multiple experimental configurations can easily be produced in the system as illustrated by the different tests performed (see specific experiments below). In addition, the approach is simple and allows for a high level of replication and throughput. In the cases described here, one of the drops corresponded to the inoculum and contained a suspension with spores (Fig. 1A-top). From this initial point, hyphae emerged and explored their surroundings in response to the trophic or biotic stimuli provided (Fig. 1A-bottom). Given the very small inoculation volume (usually 10–25 µl), one major issue that can limit the use of the system is fast desiccation. Two methods were tested to prevent the system from drying out during the experiments. For the incubation of individual Petri dishes, those were placed inside a nontreated 100 mm Petri dish with cotton filters at the bottom soaked in 2 ml of deionized water (Fig. 1B). This method was suitable if the system was to be moved or required continuous monitoring. Alternatively, an incubation chamber with moistened vermiculite at the bottom allowed the humidity of the system to be maintained at above 80% (Fig. 1C). This method was suitable for incubating several Petri dishes simultaneously and was used as the main incubation method for all the experiments with the drops. The time required by the drop to completely dry depended on the volume of the drop and the organism used. For instance, when inoculated alone, *F. oxysporum* drops remained moist for up to 1 month if kept in the humid chamber but dried out in a matter of days when left outside of the incubator.

In order to assess the reproducibility of the inoculation scheme, the final area of the drop (i.e. surface covering the bottom of the plate) was correlated to the inoculation volume. Well-defined drops (Fig. 1D) were obtained from a range of inoculation volumes between 5 and 20 µl and this was consistent for all the media tested (Fig. 1E). Although deposition was done manually, pipetting errors were negligible and the variation between multiple drops replicates was low. This was seen in the volume/area ratio of multiple individual replicates. For the duration of the experiments testing of the system (in our case 7–15 days) the optimal volume was 15–20 µl. This is mainly due to a trade-off between evaporation, which is inversely correlated to the volume, and stability of the drops during manipulation of the Petri dishes, which is more problematic with increasing volumes.

### Growth of filamentous microorganisms in the drop system and liquid movement between the drops

**Figure 2.**
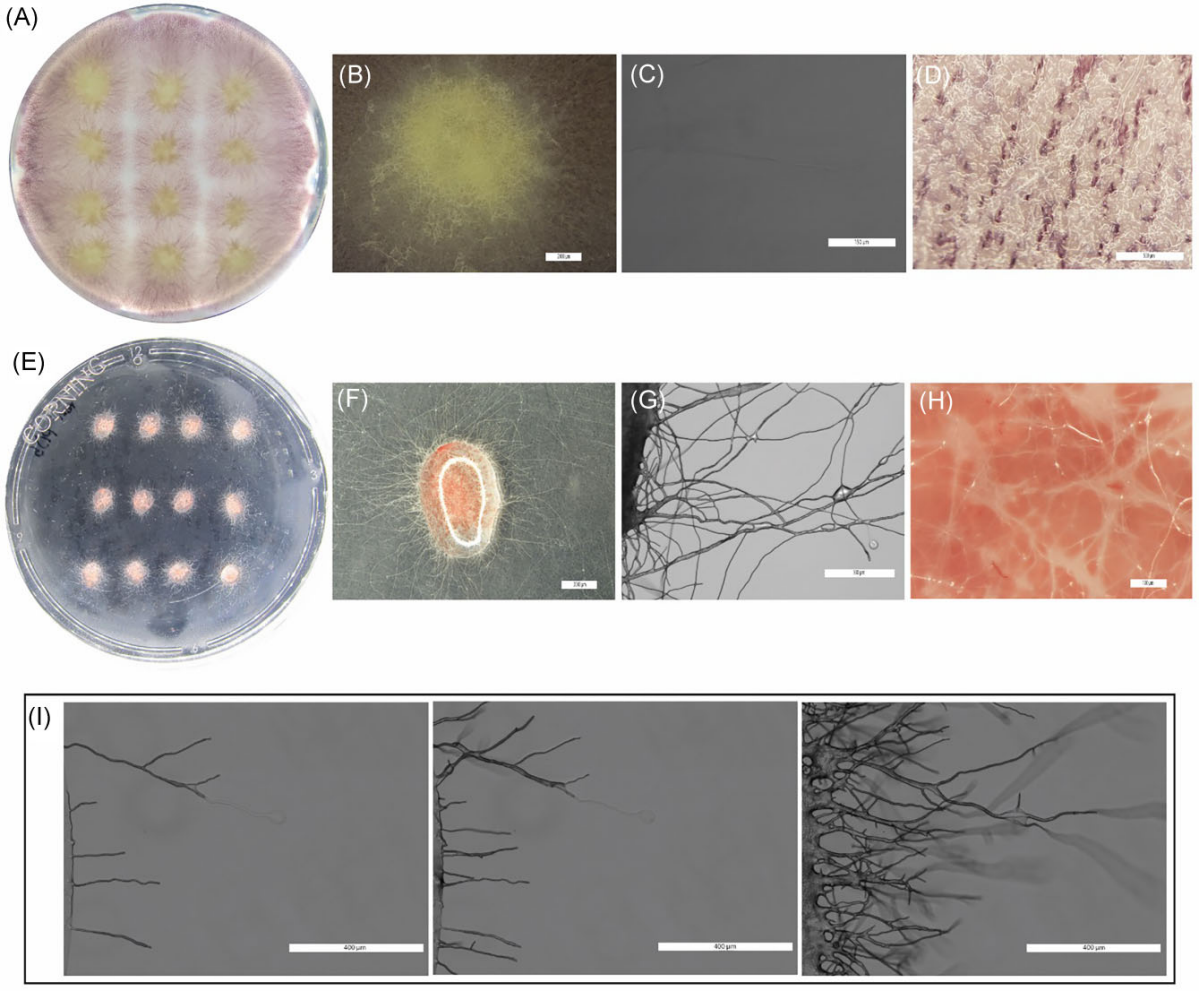
Growth of *F. oxysporum* in the drop system and comparison to normal agar media. Comparison of germination from spores and growth of *F. oxysporum* on PDA (A–D) versus germination and growth on drops with PDB (E–H). The images in (A) and (E) corresponded to the entire Petri dish (9 cm in diameter); Images (B) and (F) correspond to stereoscopic pictures of one replicate for the agar and the drop system, respectively. Images (C) and (G) correspond to images of the same area observed in (B) and (F) but taken with an inverted microscope. Images (D) and (H) show a close up of the colony surface in normal agar or in the drop system, respectively. (I) Snapshots of the supplementary video showing the growth (in MEB) of the mycelium of *F. oxysporum* out of the drop (drop edge visible on the left) in a 24-hour microscopical time lapse movie 1-day postinoculation.

Several organisms were compared to validate the performance of the fungal drops method. First, germination, growth, and exploration behaviour were assessed with *F. oxysporum* comparing the fungal drops system to normal agar plates (Fig. 2). In both systems the fungus was able to germinate, grow, and expand from multiple inoculation points (Fig. 2A and E). Images at different levels of magnification were taken to compare the performance of the two methods. In the macroscopic view of a single colony (Fig. 2B and F), single hyphae were distinguishable in the drop method but not in the solid medium. On solid media, as hyphae do not grow on a 2D plane, direct microscopy offered only limited information (Fig. 2C and D). In contrast, easy microscopic and stereoscopic observations were possible without the need for destructive sampling for the drop system, reaching a single hypha resolution (Fig. 2G and H). Furthermore, in the drop system it was possible to follow the habitat exploration strategies behaviour of *F. oxysporum* using time lapse microscopy (Fig. 2I; Supplementary Video).

**Figure 3.**
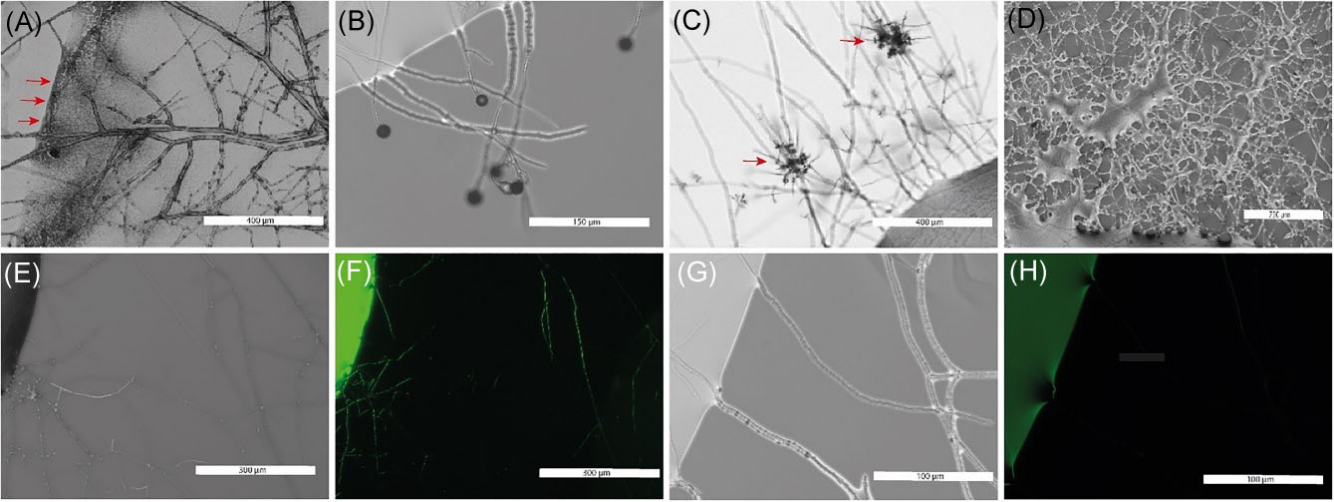
Growth of different filamentous microorganisms in and out the drops and liquid movement on the mycelium. (A) Observation of the production and deposition of a dark pigment at the edge of the drop (red arrows) by the Ascomycota *M. crassipes*. (B) Microscopical observation of *M. moelleri* exiting the drop and producing conidiophores; (C): microscopical observation of the asexual reproductive structures (conidiophores; red arrows) formed outside of the drop of the ascomycete *T. rossicum* cultivated in MEB; (D) stereoscopy image of the mycelium of the highly hydrophilic mycelium of the oomycete *P. ultimum* after existing the drop (visible on the bottom of the image). (E–H). White field and fluorescence images showing liquid redistribution by hyphae of *M. moelleri* exiting from the drop (visible on the left).

Other filamentous organisms were also all able to germinate in the drop environment and then escape from the drop to explore the surroundings (Fig. 3 A–D). Furthermore, additional mycelial characteristic could be observed for specific organisms. For instance, in the case of the ascomycete *M. crassipes*, besides the observation of multiple hyphal structures, it was possible to observe the deposition of a black pigment at the edge between the drop and the air (Fig. 3A). In *M. moelleri* (Fig. 3B) and *T. rossicum* (Fig. 3C), the exploration beyond the edge of the drop coincided with the production of asexual reproductive structures when in contact with air. The tests were not restricted to organisms of the fungal kingdom as we included the filamentous oomycete *P. ultimum*, which has previously been used as a model organism for the study of bacterial dispersal on mycelial networks (Wick et al. 2007). *Pythium ultimum* was also able to grow in and out the drops and we observed that the liquid film around the hyphae was thicker than for the other filamentous organism tested here (Fig. 3D). Moreover, with this method, a reduction of the thickness of the liquid film around the hyphae could be highlighted when *P. ultimum* was cocultured with a bacterium (Figure S2, Supporting Information). Finally, to assess whether any movement of liquid could be observed along the hyphae once the organism has exited a drop, we used fluorescein staining of the liquid phase. The comparison of white field and fluorescent images showed some fluorescent signals in hyphae outside the drops (Fig. 3E and F). However, this did not occur with all the hyphae exiting the drop (Fig. 3G and H). To test whether the signals corresponded to fluorescein being moved by the fungus existing the drop, the same experiments were conducted with *P. ultimum*. In the absence of fluorescein, fluorescent signals were observed in the mycelium, suggesting that autofluorescence can explain the fluorescence signals observed previously (Figure S3, Supporting Information). Moreover, in the presence of fluorescein, no movement of the dye was observed outside the drops, both in the presence or absence of bacteria (Figure S4, Supporting Information).

### Effect of nutrient concentration on mycelial growth of *F. oxysporum*

**Figure 4.**
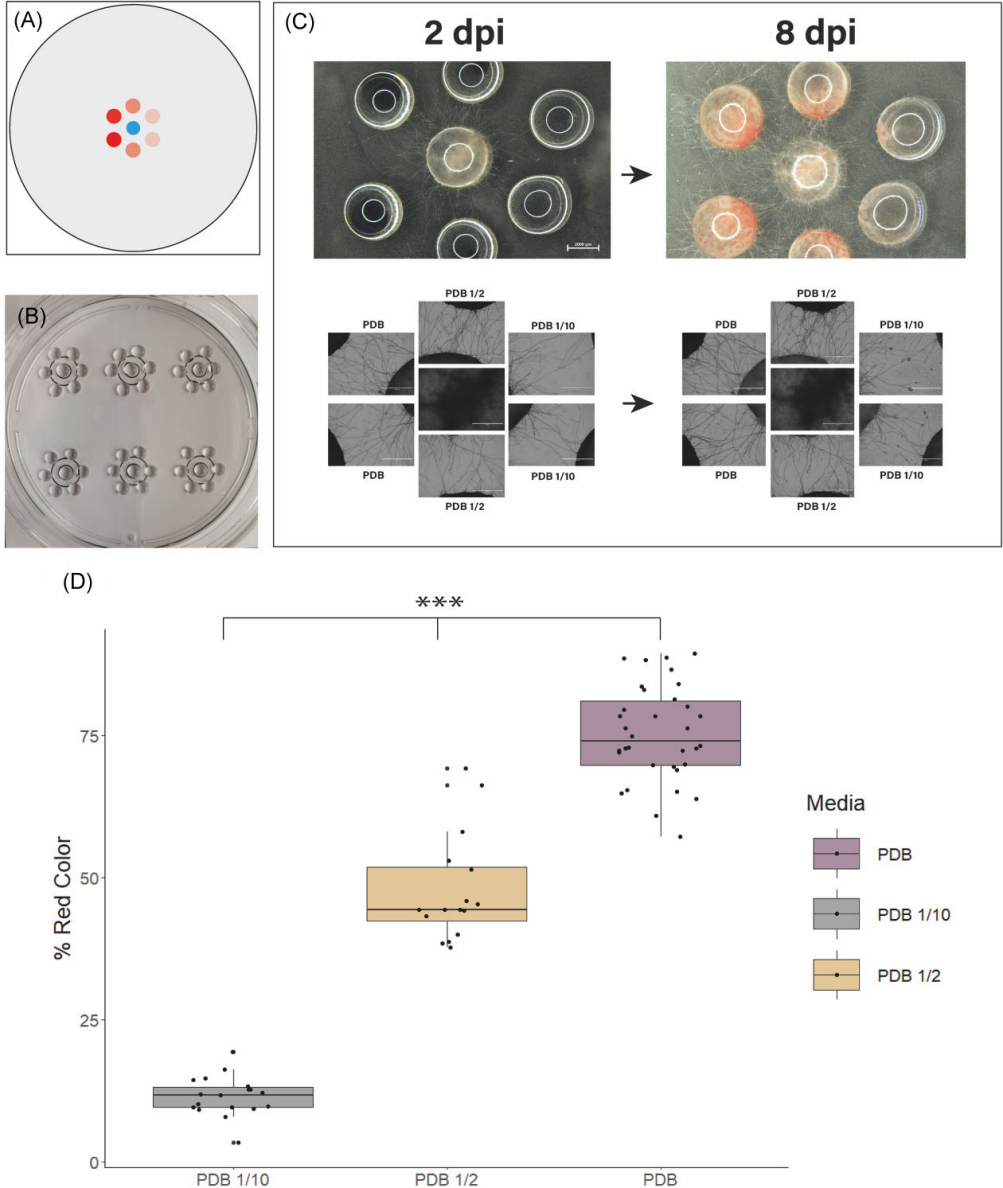
Effect of target nutrient concentration on mycelial growth. (A) Schematic representation of the flower-like disposition of drops in which the different shades of red represent different media or diluted versions of the same medium. In this case, the blue dot represents the inoculum in MEB and the red dots are technical duplicates for each medium: full strength PDB, two times diluted PDB (PDB 1/2) and 10 times diluted PDB (PDB 1/10). (B) Overview of the experiment after deposition and inoculation of six drops in a flower-like shape with a random disposition of the different target media for each flower of drops. (C) A stereoscopic overview of the inoculated system after 2 and 8 dpi is shown in the top part of panel (C). Stereoscopic images (40x) of the mycelium colonizing the different media at 2 and 8 dpi are shown on the bottom of panel (C). (D) Graphical boxplot and raw data representation of the percentage of red colour for each medium at 8 dpi (PDB violet, PDB ½ orange and PDB 1/10 in grey); for all conditions there is a statistically difference regarding the red colouration. Scale bars in (B) represent 15 mm; (C) (macroscopic pictures) 2 mm, (C) (microscopic pictures) 1 mm.

To illustrate the use of the approach to investigate questions relevant to the biology of filamentous microorganisms we investigated the effect of variable concentration of nutrients on the habitat exploration strategies of an expanding mycelium. For this, a flower-like spatial arrangement was adopted inspired by previous studies performed to test the recruitment of entomopathogenic nematodes by ravaged plants (6-arm olfactometer) (Rasmann et al. 2005). This flower-like design allows for multiple stimuli to be tested at the same time (Fig. 4A and B). Macroscopic changes in the colonies were observed and those were correlated with quantitative differences in the microscopic architecture and density of the mycelium. The mycelium of *F. oxysporum* was able to exit the inoculum drop and colonize all of the adjacent target drops (Fig. 4C). The exploratory behaviour of the emerging hyphae was already visible at 2 days postinoculation (dpi) with higher density towards the two times diluted medium (PDB 1/2; Fig. 4C). The difference in the hyphal density connecting drops with different media concentrations became more apparent at the macro and microscopic levels after 8 dpi (Fig. 4C). Moreover, the macroscopic overview of the experiment at 8 dpi revealed a difference in the concentration of a pigment secreted by the fungus in the target media (Fig. 4C). Analysis of the percentage covered by red colour in each drop (Fig. 4D), revealed a significant difference (*P*-value < .001; df: = 2) between the three different media concentrations. The full medium (PDB) being the most coloured (mean = 75.24, sd = 8.39, *P*-value < .001), then the two times diluted with an average colouration (mean = 47.74, sd = 9.49, *P*-value < .001), and last the 10 times diluted media with a slight colouration (mean = 11.54, sd = 3.5, *P*-value < .001).

**Figure 5.**
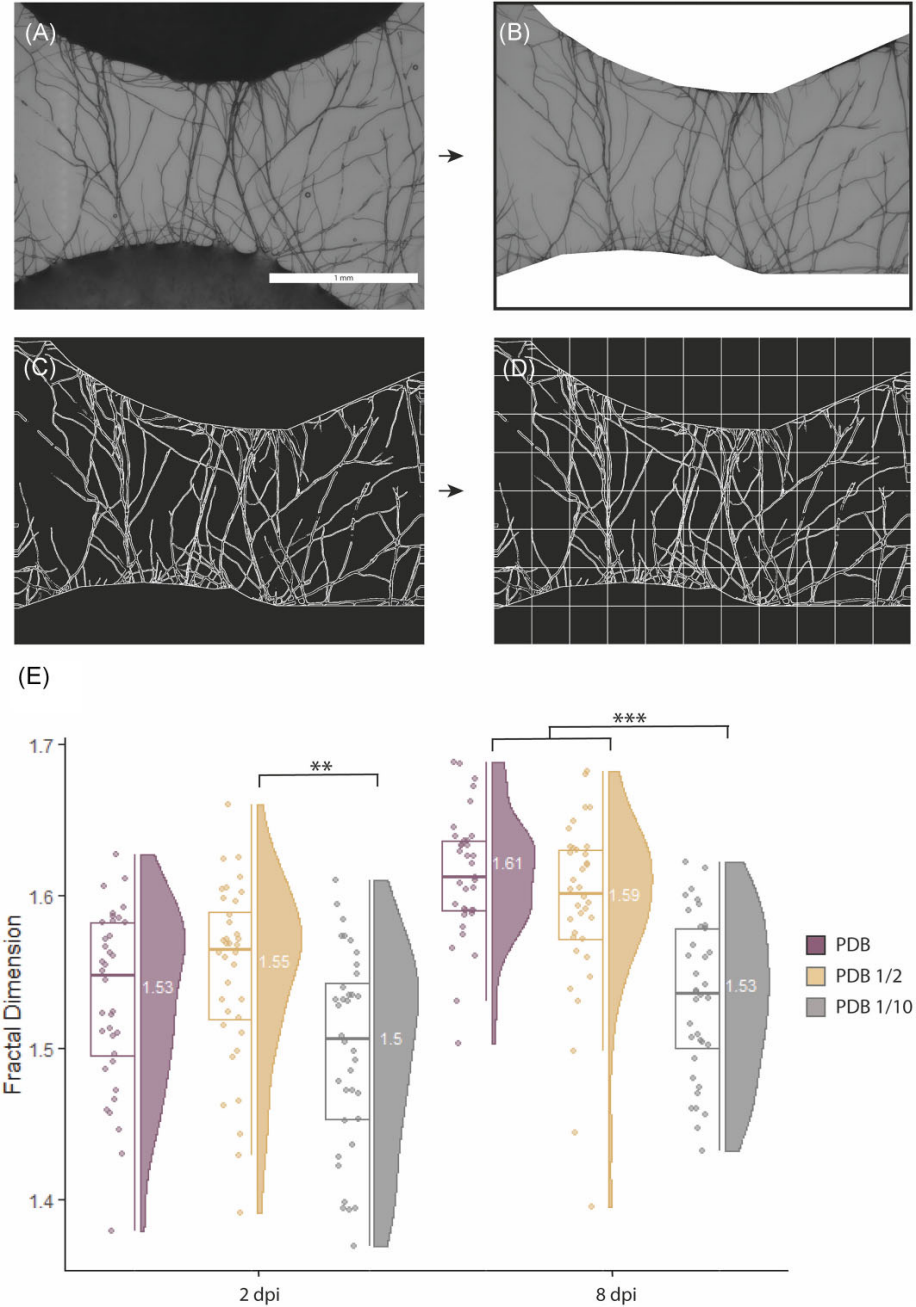
Image postprocessing and mass FD calculation to measure the effect of different nutrient concentration on the growth of *F. oxysporum* mycelium. (A) Example of an image taken between the inoculum drop (bottom) and a target drop with PDB (top) before postprocessing. (B) Same image as in panel (A) but processed using R to remove the pixel information contained in the drops and scale bar in order to obtain only the region of interest. (C) Application of the Kuwahara smoothing filter where the image is turned to black and white in order to keep only hyphae and reduce background noise. (D) Mass FD estimation using the box counting method with grids of scaling pixel sizes. (E) Raincloud plot of the mass FD estimation after image processing. The plot shows FD for each different condition tested (PDB, violet; PDB 1/2, orange; PDB 1/10, grey) after 2 and 8 dpi. For each condition the following information is highlighted: (i) raw data distribution (‘rain’) plus boxplot showing median, upper and lower quartile (left side) and (ii) data distribution (‘cloud’) labelled with the mean for each condition. At 2 dpi, the difference in mass FD between PDB and PDB 1/10 was statistically different, but not between PDB ½ and PDB 1/10. At 8 dpi, mass FD in both PBD and PBD 1/2 was statistically distinct from PBD 1/10. Pair-wise comparisons between 2 and 8 dpi were all significantly different, but the corresponding symbols were omitted from the graph for clarity reasons. For additional information see Table S2 (Supporting Information).The number of replicates for each media was 35 (PDB) or 36 (PDB 1/2 and PDB 1/10).

Mass FD was used to compare the space-filling efficiency of mycelial growth between the inoculum drop and each of the six target drops (Fig. 5). The hypothesis tested was that once the fungus had made contact with drops of different nutrient content (stimuli), it will reallocate resources to occupy more efficiently the space in which nutrient concentration is highest. After image treatment (Fig. 5A–D), mass FD was measured from pictures taken at 2 or 8 dpi. Different grid sizes were tested for the analysis in order to assess the robustness of the method and to select the best settings for our experiment. Although the absolute FD value changed with a given combination of box sizes (Table S2, Supporting Information), the overall tendency of the results is maintained, as shown by the comparison of the statistical analysis performed with different box sizes (Table S3, Supporting Information). The series of box sizes 3, 6, 12, 24, 48, 96, 192, and 384 was selected for the analysis, as the starting point (box size 3) is representative of the smallest size object that is observed in the images, and the largest size (box size 384) excludes values in which FD does not change within the images (Table S2, Supporting Information). At 2 dpi, the highest mean FD (FD = 1.549) was observed for PDB 1/2, followed respectively by PDB (FD = 1.532), and PDB 1/10 (FD = 1.498) (Fig. 5E). The highest FD at 8 dpi was found in mycelium reaching the drops containing PDB (FD = 1.613), followed by PDB 1/2 (FD = 1.593) and PDB 1/10 (FD = 1.533) (Fig. 5E). The statistical analysis (Table S4, Supporting Information) showed that there was a statistical difference in FD between 2 and 8 dpi (*F*-value = 34.43; df = 1; *P*-value = < .001), regardless of the concentration of nutrients. For all nutrient concentrations, FD was higher at 8 dpi as compared to 2 dpi (PDB: *P*-value < .001; PDB ½: *P*-value: .002 and PDB 1/10: *P*-value = .0158). Statistically significant differences between target media were also observed (F-value = 7.5823, df = 2, *P*-value < .001). Specifically, the FD at 2 dpi in PDB 1/2 (mean = 1.549) was found to be statistically different to PDB 1/10 (mean = 1.498; *P*-value = .00165). FD was also higher for PDB (mean = 1.532) compared to PDB 1/10 (mean = 1.498), but this difference was not significant (*P*-value = .05). At 8 dpi, the FD was not statistically different between PDB (mean 1.613) and PDB 1/2 (mean 1.593), but a statistical difference was found between these two and PDB 1/10 (*P*-value < .001) (Table S4, Supporting Information).

### Bacterial–fungal interactions

**Figure 6.**
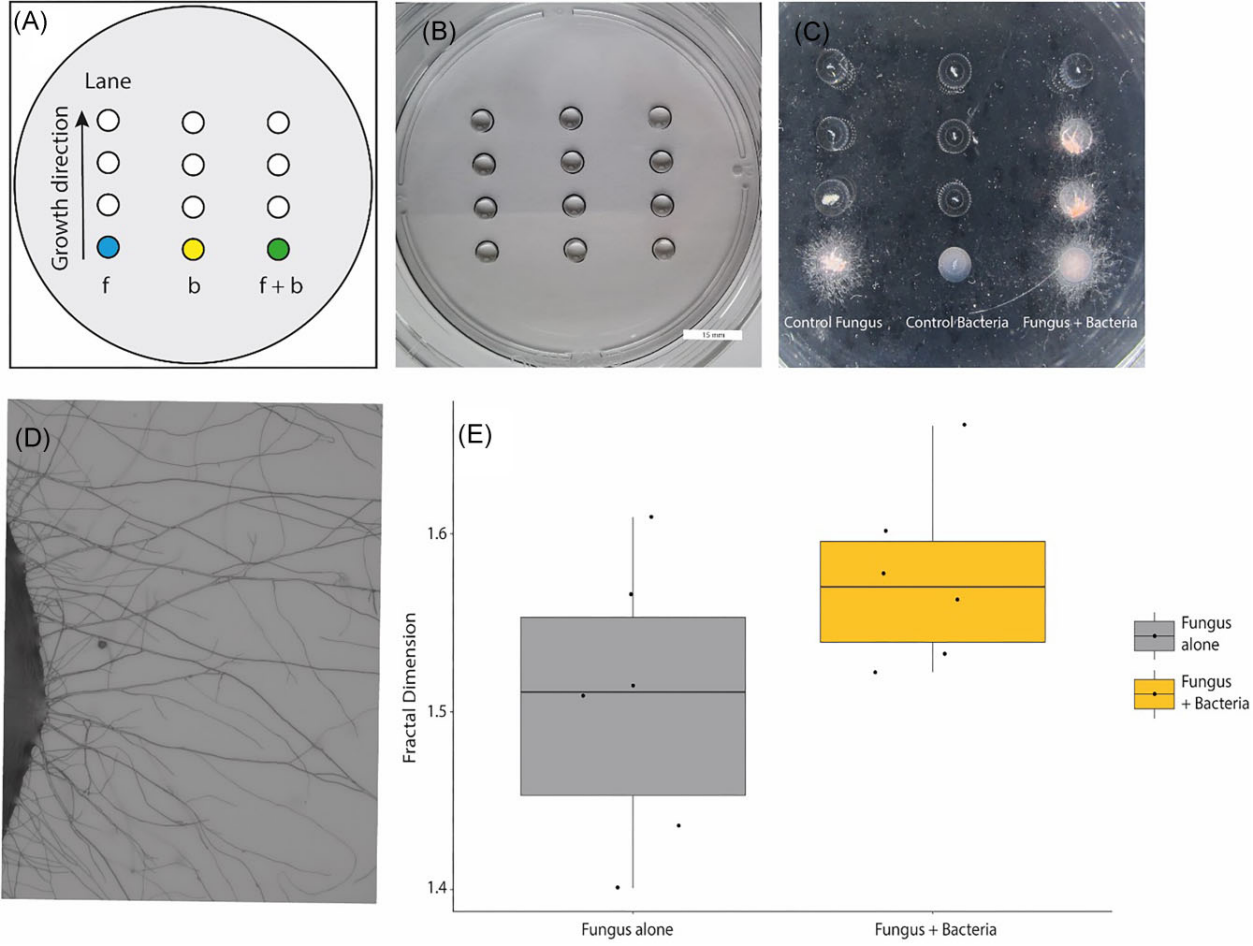
Example of a bacterial–fungal interaction. (A) Schematic representation of the system with the coloured drops representing inoculum drops at the start of a lane (vertical drops,). The arrow represents the growth direction for all conditions. The conditions corresponded to: (F) *F. oxysporum* alone (fungal control in blue), (B) *P. putida* KT2440 alone (bacterial control in yellow) and (F + B) *F. oxysporum* and *P. putida* coinoculated together (fungus + bacteria coinoculation in green). (B) Overview of the inoculated system at the start. (C) Macroscopic view of one of the systems as an example showing the effect of the bacteria on the growth and colonization pattern by the fungus (third lane vs. first lane). Picture taken after 8 days. (D) Example of images used for the calculation of the mass FD estimation. (E) Boxplot and raw data for the Mass FD estimation at 7 dpi of *F. oxysporum* alone (grey) and when coinoculated with *P. putida* (yellow). A trend towards an increasing FD in the coinoculated treatment was observed (*P*-value .08)

The system could further be used to study interactions of mycelium-forming organisms with other soil dwellers such as bacteria. To do this, a different spatial arrangement of drops was used. Specifically, multiple drops were deposited in a straight line equidistant from one another to form a ‘lane’, with the first drop containing the inoculum (Fig. 6A). Multiple lanes were placed in parallel to one another within a single Petri dish to allow direct comparison of multiple treatments. By maintaining a sufficient distance between each lane (in this case 2 cm, which is greater than the intradrop distance of 0.5 cm border to border within a lane), the successive colonization of the drops along the same lane was ensured, without interference from one lane to the next (Fig. 6B). With this setup, the effect of coculturing the bacterium *P. putida* KT2440 with *F. oxysporum* was evaluated. In a conventional confrontation experiment on MA, we observed the deposition of a red pigment by *F. oxysporum* on the bacterial inoculum. Furthermore, we observed growth of the fungus on the bacterial inoculum, after which, bacteria were no longer viable (Figure S5, Supporting Information). This prompted us to use this bacterial–fungal couple to assess the interaction in the drops method. For this, three parallel treatments (i.e. lanes) were performed per plate with the fungus inoculated alone, the bacterium alone, or both organisms in a coculture. The coinoculation with *P. putida* changed both the growth and pattern of drop colonization by *F. oxysporum*. When coinoculated with *P. putida*, the fungus was able to colonize the second drop (Fig. 6C) in five out of six replicates (Figure S6, Supporting Information). In addition, the second drop was connected to the inoculum drop at 7 dpi in the cocultures, after which the fungus continued to colonize the successive drops, reaching the last drop at 15 dpi. In contrast, when the fungus was inoculated alone, *F. oxysporum* was only able to reach the second drop at 6 dpi in three out of six replicates (Figure S6, Supporting Information). To assess if there was an effect of the bacterium on mycelium space-filling efficiency and coverage of *F. oxysporum*, a mass FD estimation on microscopic was performed on pictures taken between the inoculum drop and the first target drop for the fungus inoculated alone and when coinoculated with the bacterium (Fig. 6D). A trend in which mass FD was higher (mean = 1.576, sd = 0.050, *P*-value = .098, df = 8.57) when the two organisms were coinoculated was observed compared to the FD when the fungus is alone (mean = 1.050, sd = 0.077; Fig. 6E). Finally, to assess bacterial viability, bacterial abundance was measured by colony forming counting in the drops overtime. In the controls with bacteria only, bacterial abundance fluctuated, but bacteria were detected throughout the entire duration of the incubation with over 1 × 10^9^ cells µl^−1^ after 7 days (Figure S7, Supporting Information). In contrast, in the coinoculated drops the bacterial abundance declined after 5 days in the inoculum drop, with no detectable bacteria from day 7. Hence, as bacteria were no longer detectable in the inoculum drop, when the fungus reached the second drop bacteria were not detected to disperse to the second drop.

## Discussion

This study presents an easy-to-use approach to observe mycelial development in response to different trophic or biotic habitat conditions. This approach allows to observe different levels of organization, from mycelial colonies to individual hyphae. It is also fast and inexpensive, which allows generating numerous experimental designs with a high level of replication, minimal reagents, and using standard laboratory material. In addition to this, the drops were consistent in volume and area, ensuring highly similar conditions upon replication. Furthermore, the drops are deposited onto transparent surfaces treated for cell adhesion, thus rendering them compatible with direct microscopical observation, including fluorescence imaging. This allows the use of an inverted microscope in a nondestructive manner and without excessive manipulation enabling *in situ* observations. Finally, this method offers the opportunity for the production of time lapse movies allowing to assess the dynamics of fungal growth and response to different factors affecting exploration and growth on filamentous microorganisms.

The drops are placed on a cell-treated dry surface with a constant distance between them, mimicking an artificial 2D soil in which different stimuli surround a propagule of an organism. In this way, the drops recreate a situation in which an organism (represented by either as a spore or as a hyphal fragment) is confronted to an environment with a limited amount of nutrients, and thus is forced to explore the surroundings to find additional resources. Previous studies have shown the complex behaviour of filamentous fungi when thriving in the vast and heterogeneous niches in soils (Griffin 1972, Ritz and Young 2004), or when confronted with other organisms (Fricker et al. 2007, Wrzosek et al. 2016, Deveau et al. 2018, Hiscox et al. 2018). The conclusions of these studies are based on macroscopic observations of mycelia grown on agar/wood blocks (Boddy 1993) or by visualizing single hyphae with microfluidic devices (Schmieder et al. 2019). However, the simultaneous observation of mycelia at the macro and microscopic levels is hard to achieve in either of these approaches. This is one of the most significant advantages offered by the drop system. The combined macro- and microscopic analyses allow to bring new insights into how filamentous fungi manipulate their network architecture, something essential not only for survival and colonization in soil ecosystems (Nannipieri et al. 2003, Delgado-Baquerizo et al. 2016), but also in other ecosystems (Adrio and Demain 2003). Our approach, even if still far from a realistic reconstruction of a natural soil as compared to other methods (Boddy 1993, 2000), represents a simple and well-controlled microbial ecosystem in 2D. Compared to other methods, the nutrient supply is limited by the size of the drops, as compared to normal agar plates where the initial nutrient content is higher, or in microfluidics where a nutrient flow can be maintained. However, this allows for a precise control of the nutrient content and heterogeneous availability. This could be a better representation of a nonsaturated soil in which nutrients may be spaced, diverse, and limited.

The different tested organisms were all able to colonize and exit the drop (Fig. 2). Furthermore, we were able to observe additional features such as the formation of asexual reproductive structures in *T. rossicum* and *M. moelleri* and the production and deposition of melanin by *M. crassipes* (Fig. 3). The method should be of wide application with different media and conditions for specific organisms. The drops are mostly connected by the mycelium and its activity, and not by the liquid film formed on them, as little to no movement of the media was observable on the mycelium, and no fluorescence could be detected in the target drop in the experiments with fluoresceine. However, when different media and condition are tested, a control should be performed to validate this observation under different experimental conditions to avoid artefacts.

The usefulness of the fungal drop approach to study fungal biology was illustrated using two examples. In the first one, the approach was used to collect qualitative and quantitative data regarding mycelial growth in response to nutrient availability (Fig. 4). At the macroscopic level differences in mycelial pigmentation related to different nutrient concentrations were observed and the changes in colour were assessed quantitatively by image analysis. At the microscopic level, space-filling efficiency was measured quantitatively using mass FD analysis (Fig. 5). Our results with *F. oxysporum* demonstrate that network complexity and space-filling efficiency increased significantly when the mycelium connected patches with higher nutrient content (PDB; PDB1/2). This suggests that the fungus coordinates its network to increase efficiency with increasing nutrient conditions, as proposed in previous studies (Veresoglou et al. 2017). The increased network complexity might be the result from regrowth of the fungus from target drops towards the inoculation drops. However, this could not be controlled here. Moreover, the increasing difference in FD after 8 dpi supports the idea that fungi display learning and decision-making capabilities (Aleklett and Boddy 2021). In the future, other measurements of network complexity could be tested on the images generated by the method (Heaton et al. 2012).

Soil is not only composed of a heterogeneous matrix of nutrients, but it is also inhabited by an enormous number of living organisms (Fitter et al. 2005, Aleklett et al. 2018). Biotic interactions, hence are key in ecological processes including pollutant degradation and nutrient turnover (Kohlmeier et al. 2005, Khan et al. 2023). Therefore, the use of the approach was also illustrated by assessing multispecies interactions. Using the drop system, it was possible to observe the effect of bacteria on the colonization of a new habitat by the fungus *F. oxysporum* (Fig. 6). Fungal colonization was more effective in the cocultures, but it did not correlate with the colonization of the new drop by bacteria. Instead, bacterial populations collapsed prior to the colonization by the fungus of the new drop (Figure S7, Supporting Information). In this case, dead bacteria might be an additional nutritional source for the fungus (Barron 1988, Pion et al. 2013), which may be used to improve exploratory behaviour. This is supported by the observations showing that in the coinoculation condition the fungus was better able to reach a second drop, as compared to the control with the fungus alone (Fig. 6; Figure S7, Supporting Information). Furthermore, mass FD analysis between the first and second drop showed a trend towards higher complexity and coverage for the mycelium in the coinoculation treatment, compared to the conditions in which the fungus was inoculated alone. This suggests that the interaction was beneficial for the fungus in terms of environmental colonization. Another hypothesis may be that bacteria produced growth factors for the fungus, something that would explain the higher growth of the fungus when cocultured with *P. putida*, but it does not explain the death of the bacteria. Other studies have shown that *Pseudomonas* spp. can be used as a biocontrol agent against *Fusarium* wilt in plants (Tari and Anderson 1988, Bora et al. 2004). Although this suggests a possible negative relationship between the two organisms, this is likely highly strain specific and the outcome might be determined by the environment in which the interactions take place. In our experiment, bacteria appear to act as an additional nutrient source in agreement with the observations of the first experiment in which higher FD was correlated to higher nutrient content; and by the mycelial growth on the bacterial inoculum when the two organisms are confronted on conventional agar media (Figure S5, Supporting Information). However, further experiments are required to test either hypothesis. Combining the drop system with methods such as proteomics or metabolomics could provide further insights into the mechanisms behind these observations. The combination of our approach with further improvements in the multiomics methods would certainly contribute to a better mechanistic understanding of the interactions observed here.

## Conclusion

Given its simplicity, our approach could be used in the future to observe additional types of interactions in microorganisms with a filamentous growth (e.g. predation). Moreover, this approach can allow visualizing the specific areas of production and deposition of other important secondary metabolites in a complex mycelial network. Thanks to the possibility of generating time-lapse imaging coupled with fluorescent tagging, the system could enable the observation of complex fungal behaviours such as mating fusion between compatible strains. The ability to build a 2D heterogenous environment connected by a fungal network could become a very important tool to study bacterial–fungal interactions, as well as the interaction of fungi (and fungi-like microorganisms) with other soil organisms. In conclusion, the examples provided demonstrate how the drop system represents a valuable tool to study fungal biology, while maintaining low costs and requiring minimal expertise. This method, coupled with image analysis, provides new insights into the study of fungal behaviour macroscopically and at a single-hypha level. This approach complements in this way the use of more complex experimental platforms such as microfluidic devices.

## Supplementary Material

uqad042_Supplemental_Files
