# Supplementary material for: Fungal drops: a novel approach for macro- and microscopic analyses of fungal mycelial growth
Source: Microlife. 2023 Oct 18;4:uqad042. doi: 10.1093/femsml/uqad042 (PMC10642649; doi:10.1093/femsml/uqad042)
Supplement: uqad042_Supplemental_Files [file uqad042_supplemental_files.zip › sporedrops_MS_SI_14sept2023.docx]

# **Fungal drops: a novel approach for macro- and microscopic analyses of fungal mycelial growth**

# Authors: Matteo Buffi^1^, Guillaume Cailleau^1^, Thierry Kuhn^1,2^, Xiang-Yi Li Richter^1,2^, Claire E. Stanley^3^, Lukas Y. Wick^4^, Patrick S. Chain^5^, Saskia Bindschedler^1^*, Pilar Junier^1^*

Affiliations: 1. Laboratory of Microbiology, University of Neuchâtel, Neuchâtel, Switzerland. 2. Laboratory of Eco-ethology, University of Neuchâtel, Neuchâtel, Switzerland; 3. Department of Bioengineering, Imperial College London, London, United Kingdom. 4. Helmholtz Centre for Environmental Research, Department of Environmental Microbiology, Leipzig, Germany. 5. Bioscience Division, Los Alamos National Laboratory, Los Alamos, New Mexico, USA.

# *Co-corresponding authors: [saskia.bindschedler@unine.ch](mailto:saskia.bindschedler@unine.ch); [pilar.junier@unine.ch](mailto:pilar.junier@unine.ch)

# Running title (50 characters): Observation of fungal mycelial growth

Keywords (6): Bacterial-fungal interactions, mycelium observation, quantification fractal dimension, fungal highways, mycological method, user-friendly

Journal FEMS µlife

**Supplementary Material**

Supplementary Information Table S1. Media used in this study.

Supplementary information Table S2: Example for the selection of the good box sizes for the Estimation of Fractal Dimension: On top, as an example, six different images were analysed (Label) using a box size of 3, 6, 12, 24, 48, 96, 192, 384, 768, 1536. In red (D10) the FD obtained by considering the counts of all box sizes (3 – 1536), In grey (D9) FD obtained by considering only the first 9 box sizes (3 – 768) and in blue (D8) FD obtained by considering the first 8 box sizes (3 – 384). Below, the mean FD values for multiple conditions is reported. For our analysis we decided to consider only the columns in which the number of counted boxes differed between samples (in this example, box size 384), however, the values calculated for 9 or 10 box sizes (D9 and D10) show the same tendency. In SI Table S3 it is possible to observe that the changes in the calculated FD values does not affect the statistical significance of the results obtained in the experiment.


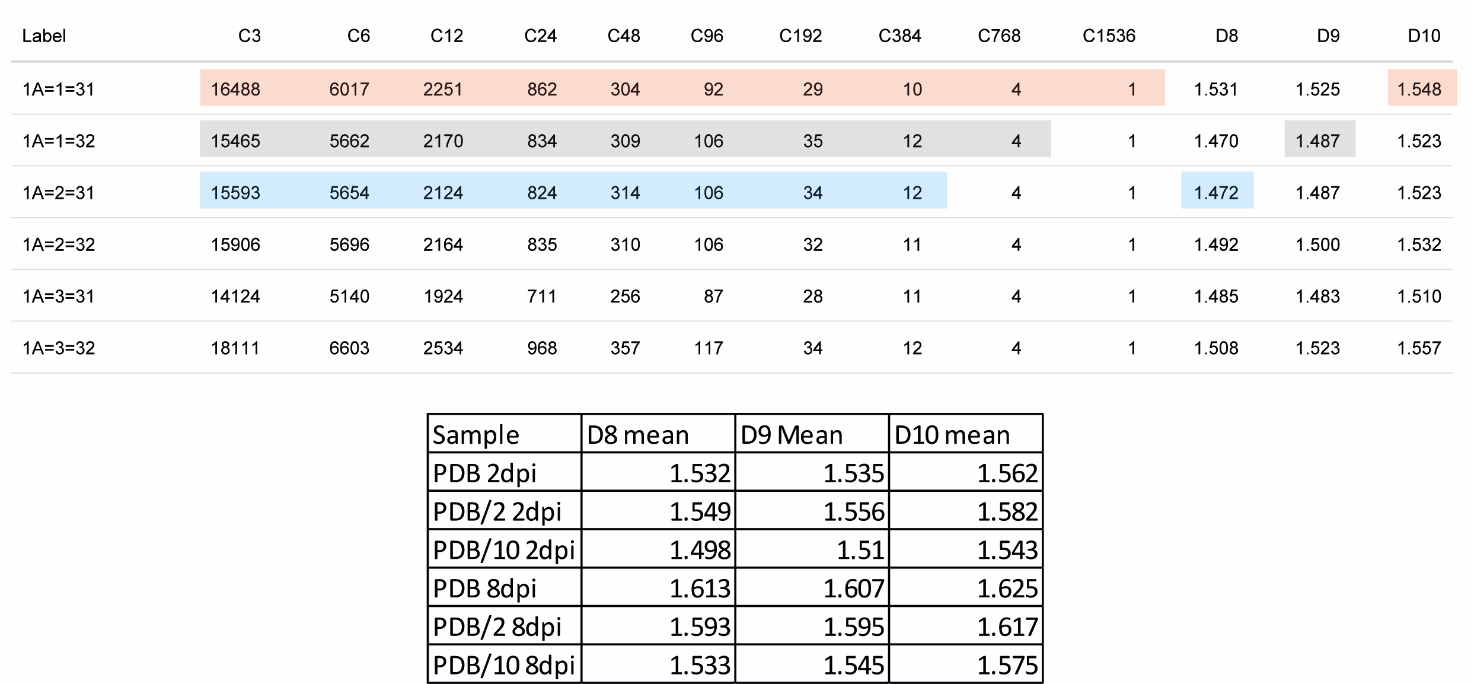


Supplementary information Table S3: Different p-values obtained when comparing the statistical significance of the differences in fractal dimension measured using different box sizes for the box counting method. This comparison shows the robustness of the statistical significance for the comparisons performed within a single experiment.

| **Box sizes**  **Comparisons** | **1, 2, 4, 8, 16, 32, 64, 128, 256, 512** | **1, 2, 4, 8, 16, 32, 64, 128, 256** | **1, 2, 4, 8 16, 32, 64, 128** | **3, 6, 12, 24, 48, 96, 192, 384, 768** | **3, 6, 12, 24, 48, 96, 192, 384** |
| --- | --- | --- | --- | --- | --- |
| **2dpi – 8dpi** | < 0.001 *** | < 0.001 *** | < 0.001 *** | < 0.001 *** | < 0.001 *** |
| **PDB 2dpi – PDB 8dpi** | < 0.001 *** | < 0.001 *** | 0.00741 ** | < 0.001 *** | < 0.001 *** |
| **PDB 1:2 2dpi – PDB 1:2 8dpi** | < 0.001 *** | 0.005 ** | 0.005 ** | 0.0043 ** | 0.002 ** |
| **PDB 1:10 2dpi – PDB 1:10 8dpi** | 0.0158 * | 0.0356 * | 0.106 | 0.00931 ** | 0.0158 * |
| **Target media** | 0.002 ** | < 0.001 *** | 0.001257 ** | < 0.001 *** | < 0.001 *** |
| **2dpi** |  |  |  |  |  |
| **PDB – PDB 1:2** | 0.33 | 0.3412 | 0.384 | 0.24 | 0.468 |
| **PDB – PDB 1:10** | 0.15 | 0.0983 | 0.085 | 0.163 | 0.05 |
| **PDB 1:2 – PDB 1:10** | 0.00413 ** | 0.0020 ** | 0.002 ** | 0.002 ** | 0.00165 ** |
| **8dpi** |  |  |  |  |  |
| **PDB – PDB 1:2** | 0.3 | 0.259 | 0.184 | 0.55 | 0.237 |
| **PDB – PDB 1:10** | < 0.001 *** | < 0.001 *** | < 0.001 *** | < 0.001 *** | < 0.001 *** |
| **PDB 1:2 – PDB 1:10** | < 0.001 *** | < 0.001 *** | < 0.001 *** | < 0.001 *** | < 0.001 *** |

Supplementary information Table S4: Results Fractal dimension estimation results coming from a two factor ANOVA and a post hoc-Tukey contrast for pair-wise comparison between the different factors. In a second table the Means and standard deviation for the FD value obtained. Box size used: 3, 6, 12, 24, 48, 96, 192, 384

| Comparison | F-value | Degree of Freedom | p-value |
| --- | --- | --- | --- |
| 2dpi – 8dpi | 34.4397 | 1 | < 0.001 *** |
| PDB 2dpi – PDB 8dpi |  |  | < 0.001 *** |
| PDB 1:2 2dpi – PDB 1:2 8dpi |  |  | 0.002 ** |
| PDB 1:10 2dpi – PDB 1:10 8dpi |  |  | 0.0158 * |
| Target media | 7.5823 | 2 | < 0.001 *** |
| 2dpi |  |  |  |
| PDB – PDB 1:2 |  |  | 0.468 |
| PDB – PDB 1:10 |  |  | 0.05 |
| PDB 1:2 – PDB 1:10 |  |  | 0.00165 ** |
| 8dpi |  |  |  |
| PDB – PDB 1:2 |  |  | 0.237 |
| PDB – PDB 1:10 |  |  | < 0.001 *** |
| PDB 1:2 – PDB 1:10 |  |  | < 0.001 *** |

| **Connection** | **Mean FD** | **Standard Deviation FD** |
| --- | --- | --- |
| **PDB 2dpi** | 1.532 | 0.057 |
| **PDB 1:2 2dpi** | 1.549 | 0.059 |
| **PDB 1:10 2dpi** | 1.498 | 0.064 |
| **PDB 8dpi** | 1.613 | 0.04 |
| **PDB 1:2 8dpi** | 1.593 | 0.059 |
| **PDB 1:10 8dpi** | 1.533 | 0.052 |

*Supplementary Figures Legends:*

Figure S1: Masks used to place the drops for the different experiments. (A-B) Bacterial fungal interactions, dotted lines are used to keep the drops at a constant distance. (C) Choice of nutrients. Masks were created with Adobe Illustrator®

Figure S2: Comparison of the thickness of the liquid film surrounding hyphae of *Pythium ultimum* when grown alone (A-D) or co-inoculated with *Pseudomonas putida* KT2440 (E-H). Two close-up images (D and H) show the details of the variable thickness of the liquid film.

Figure S3: Evaluation of autofluorescence in the mycelium of *Pythium ultimum* when grown alone (A) or co-inoculated with *Pseudomonas putida* KT2440 (B). Images taken at different magnifications.

Figure S4: Evaluation of the movement of liquid from the drop on the mycelium of *Pythium ultimum* using fluorescein. (A) *Pythium ultimum* grown alone. (B) *P. ultimum* in co-inoculated with *Pseudomonas putida* KT2440. Images taken at different magnifications.

Figure S5: Confrontation assay on MA between *Fusarium oxysporum* and *Pseudomonas putida* KT2440. On the left the MA Petri dish with *F. oxysporum* inoculated in the centre and the inoculum with *P. putida* on top. The two organisms were inoculated at the same time and the image shows the interaction once the fungal growth front has reached the bacterial inoculum. The image on the right corresponds to a magnification of the bacterial inoculum area showing the overgrowth of *F. oxysporum* on bacteria. Upon fungal colonization bacteria viability was tested by trying to re-culture the red inoculum on NA with cycloheximide (500mg/L), but the bacteria were shown to be dead.

Figure S6: Overview of the individual replicates in the experiment testing fungal-bacterial interactions presented in Figure 4. For each replicate (3 drop lanes): first lane is the fungus inoculated alone, second line the bacteria inoculated alone and in the third line the fungus and the bacteria co-inoculated together.

Figure S7: Counting of viable bacteria over time. The grey line corresponds to the counting of viable bacteria in the control inoculated with bacteria only. The orange line corresponds to the counting of viable bacteria from drops that were co-inoculated with *F. oxysporum*. Error bars represent the standard deviation between three replicates.


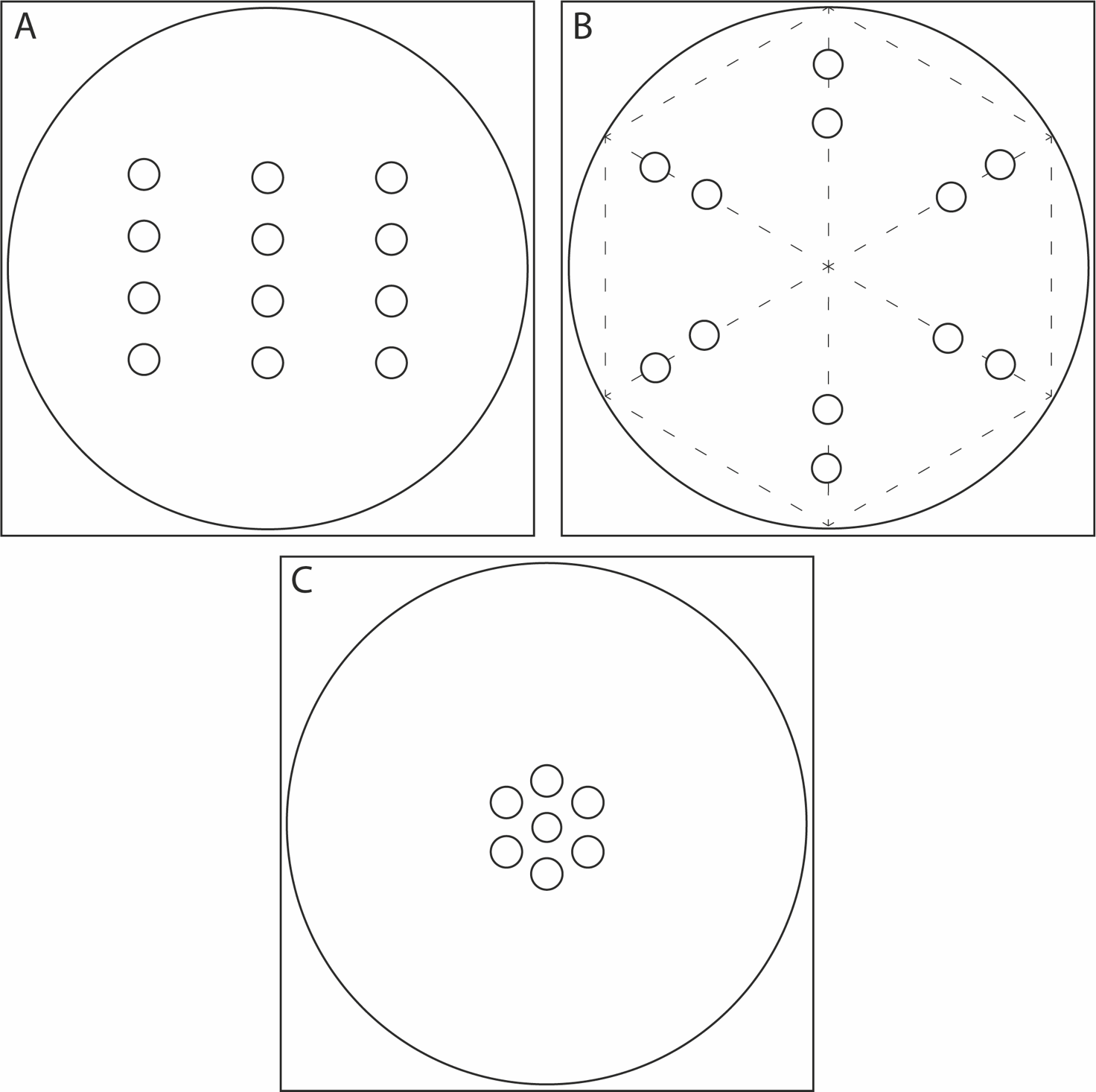


Figure S1


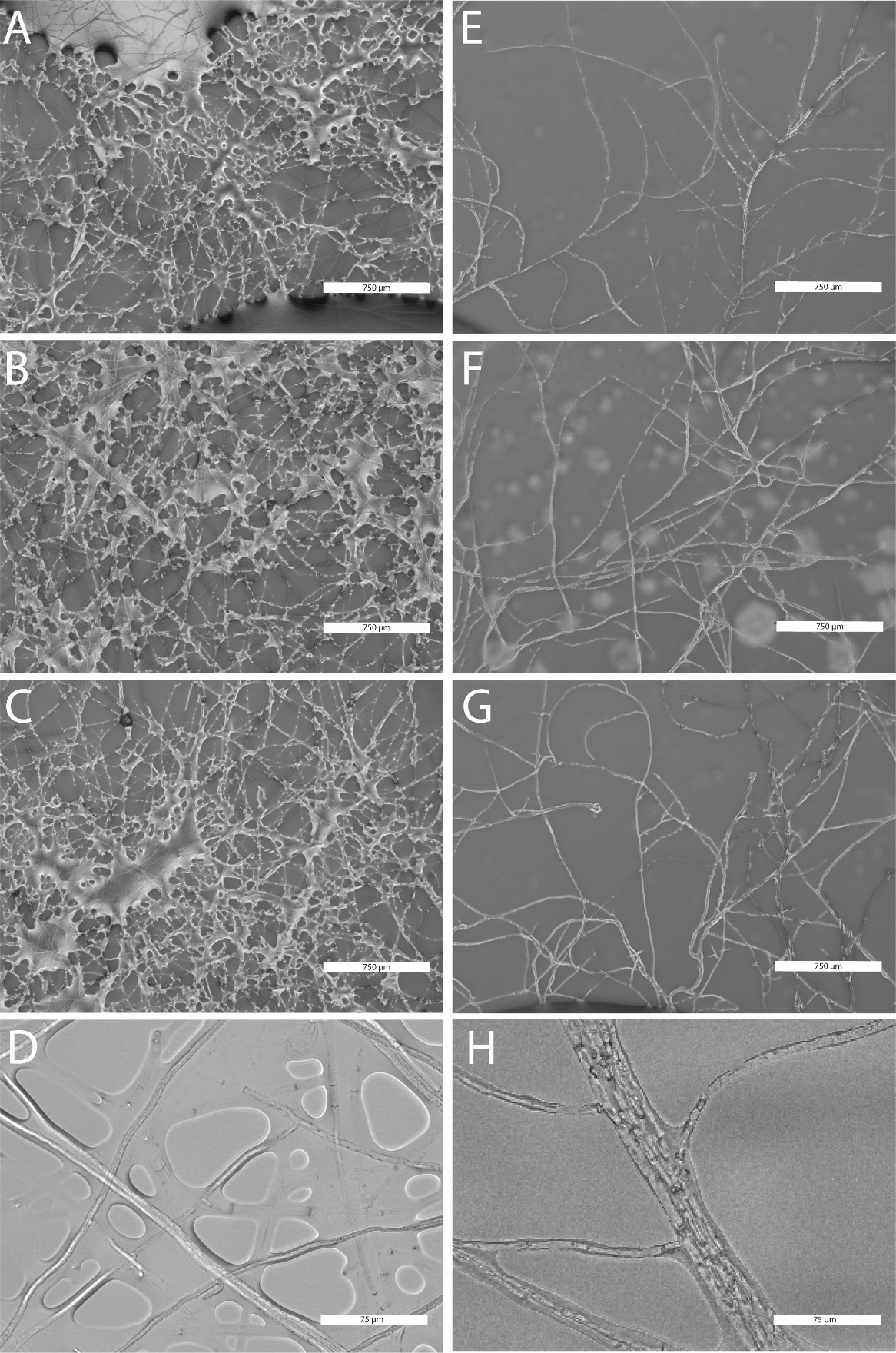


Figure S2


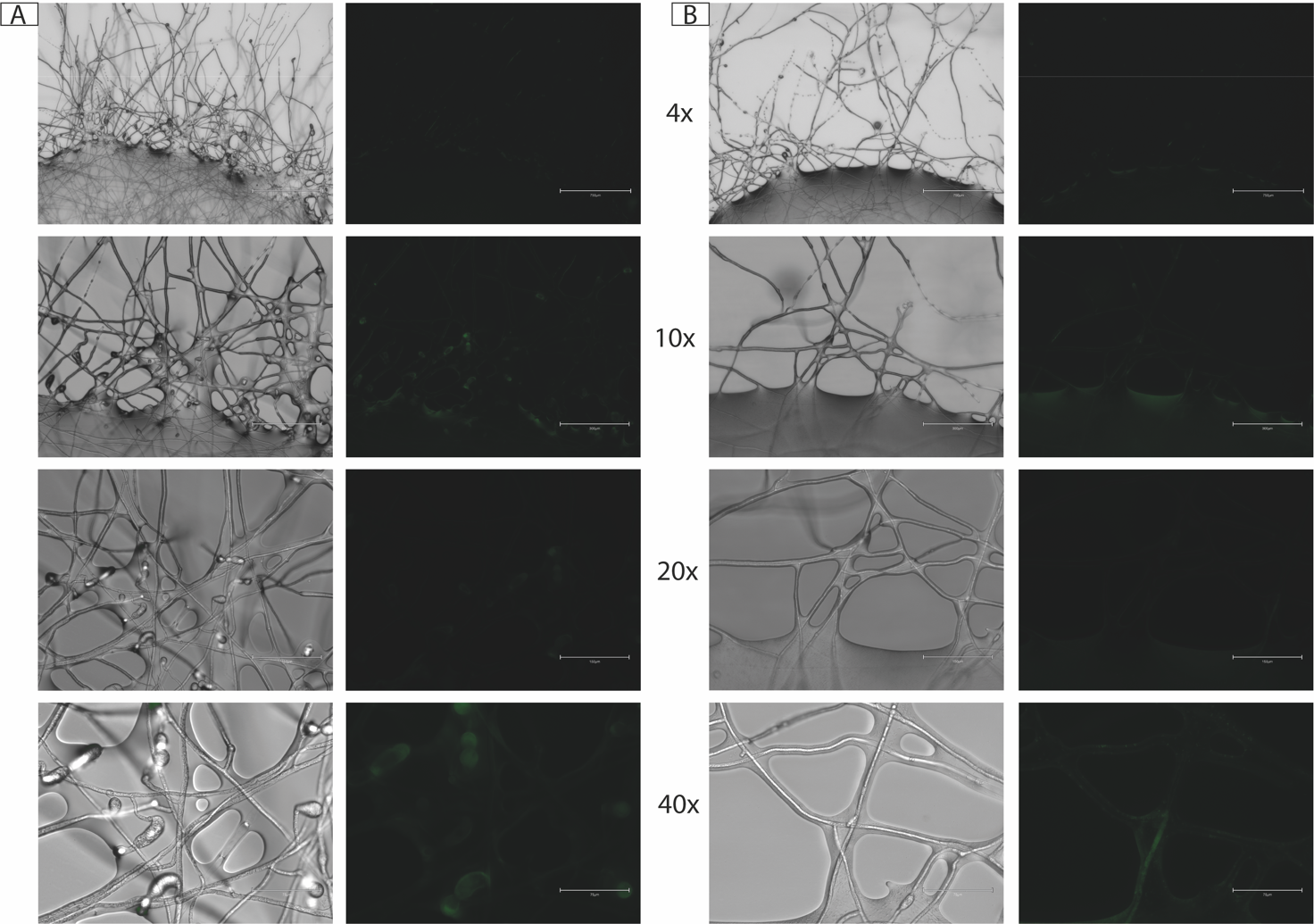


Figure S3


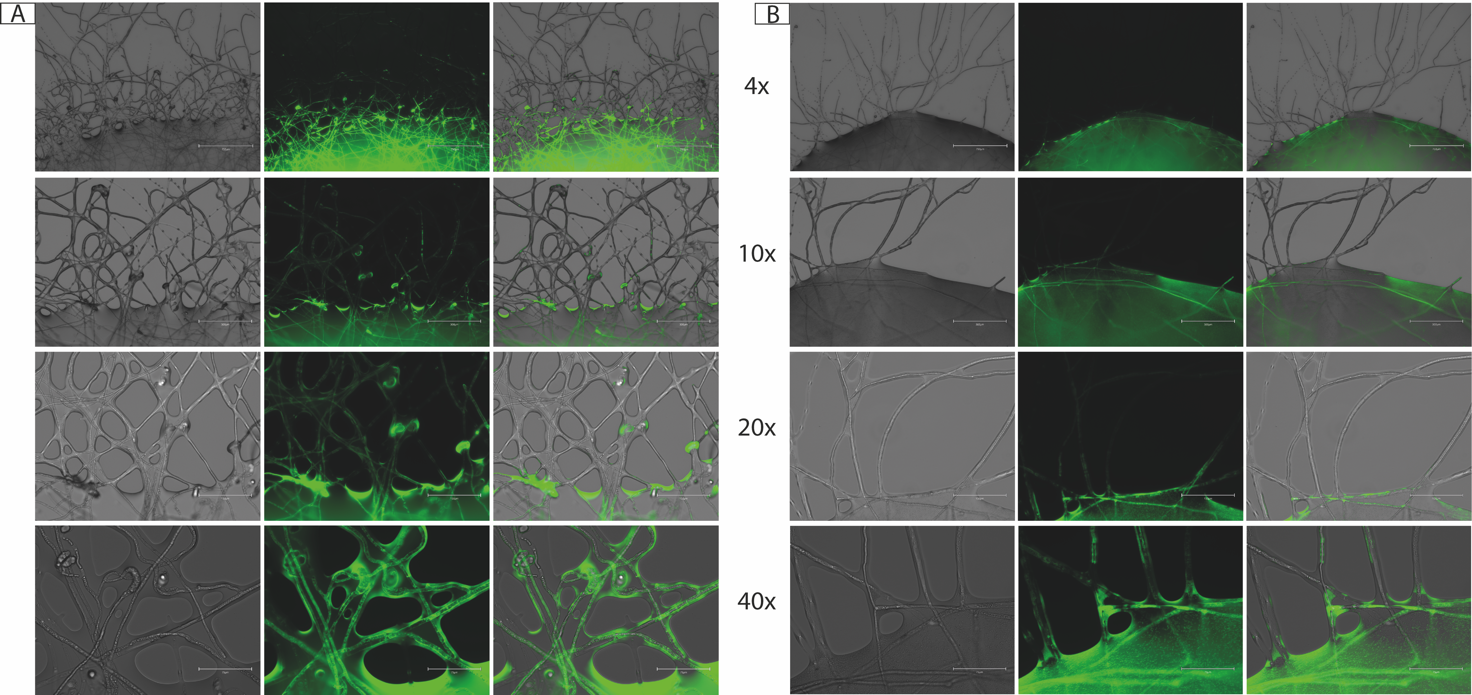


Figure S4


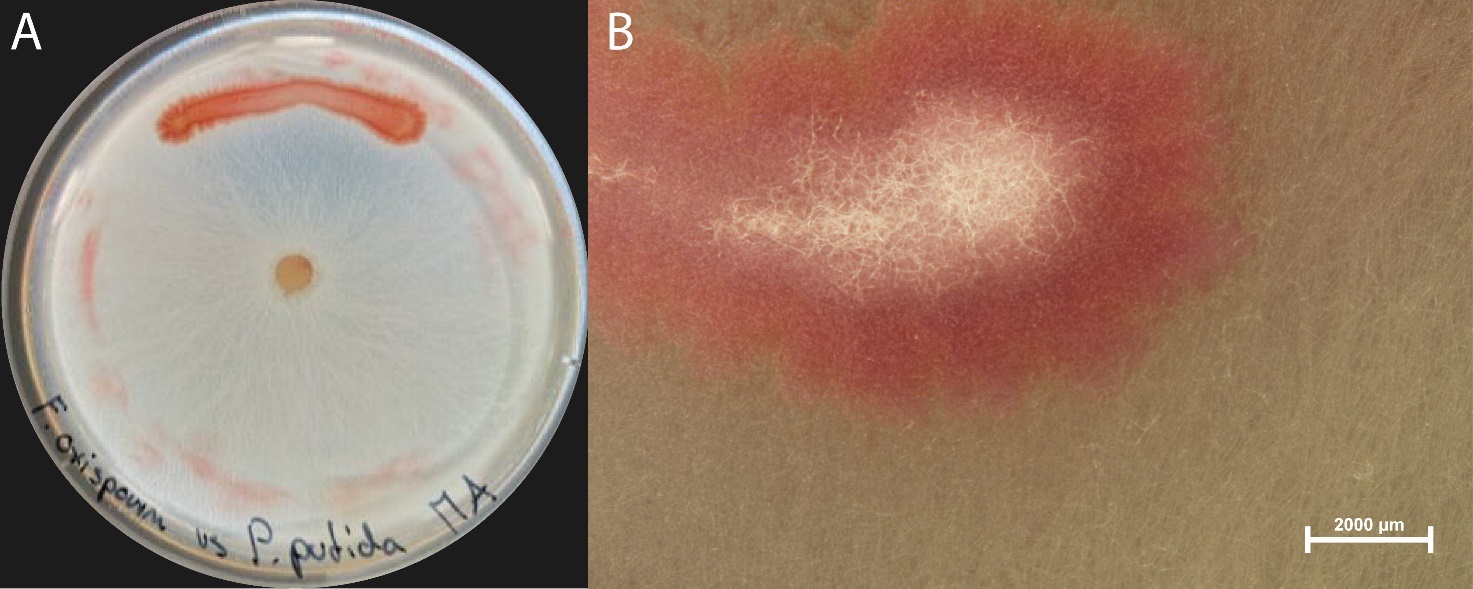


Figure S5


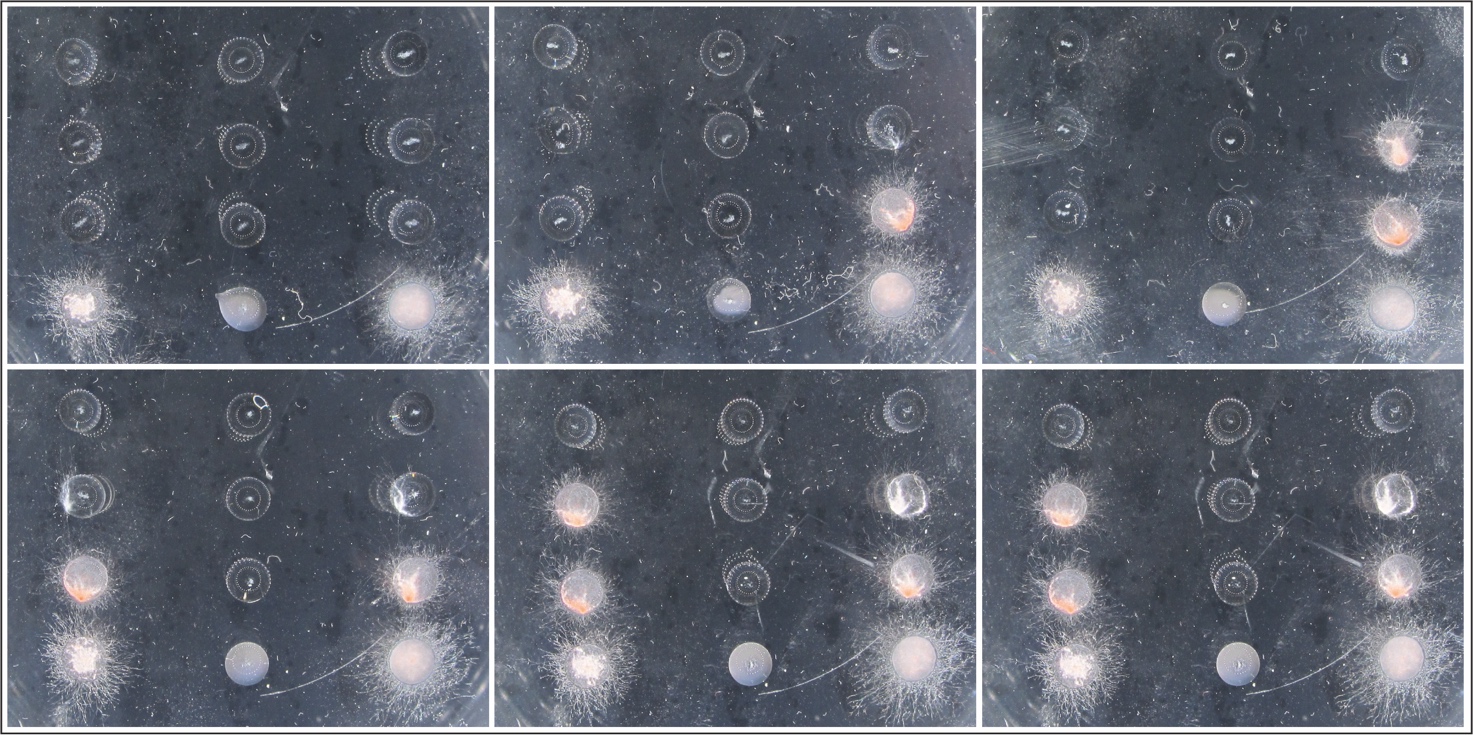


Figure S6


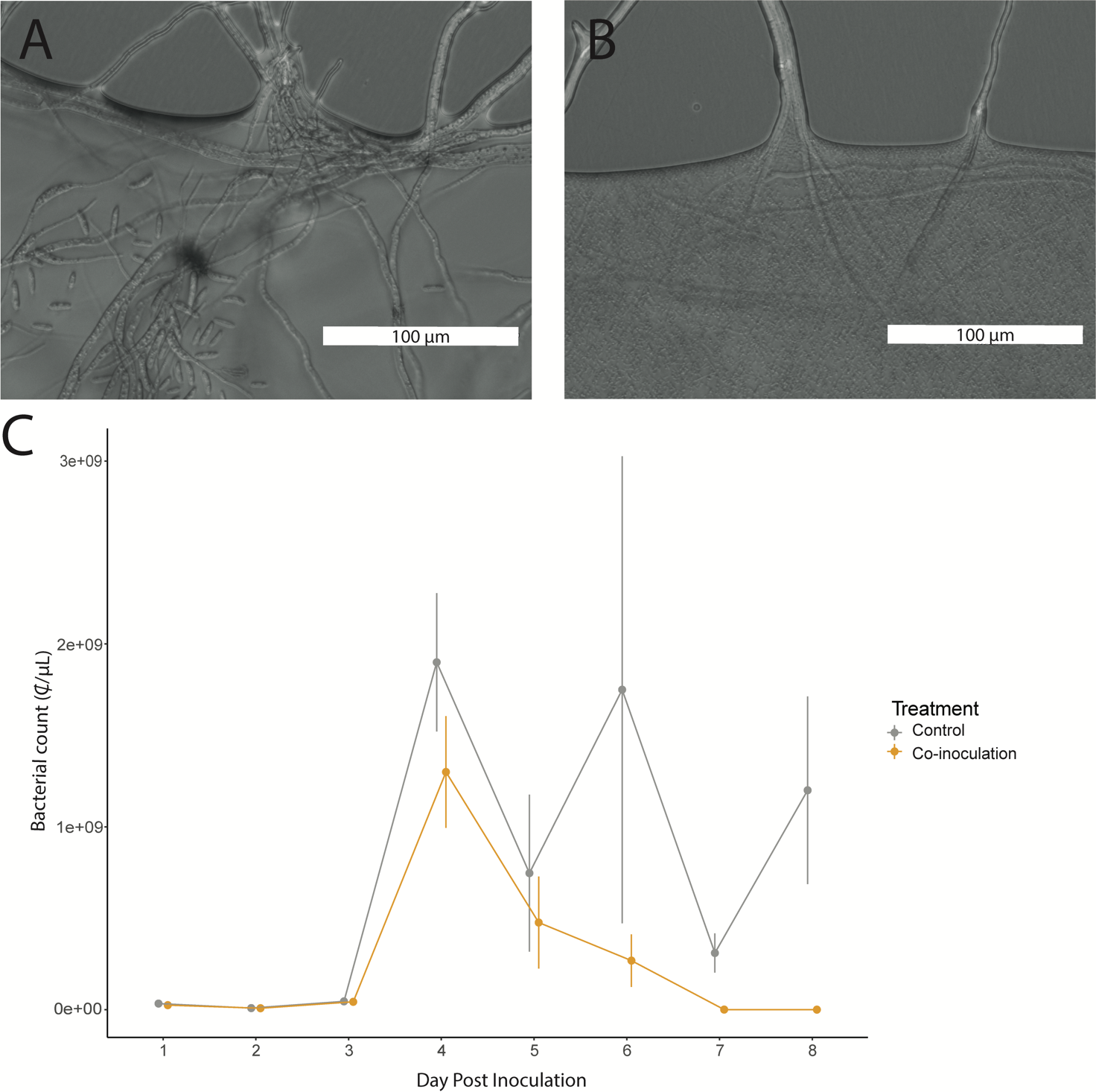


Figure S7

Supplementary Information

**Image preparation in R**

In order to remove the droplet in the microscope images we performed a two-modules treatment in R (R Core Team, 2017) using the scripts provided below.

**Step 1: droplet removal preparation step**

The first module, using the *png_crop_by_polygon_clean.R* script produces a hand-made polygon aiming at cropping each image (by clicking on the displayed image to exclude the droplet from the polygon) in a source folder (here 12 images per modality). This creates for each image treated a raster image, result of the cropping. These data are then stored for further use.

**Step 2: droplet removal step**

The second module, using the *Script_2crop_clean.R* script aims at cleaning the original image by importing the cropped raster previously created. This module sets the cropped area in pure white for easing further computation in ImageJ. These two modules are supported by the packages *imager* (Barthelme, 2020) and *magick* (Ooms, 2020) for image computation and *sp* (Pebesma and Bivand, 2005) and *raster* (Hijmans, 2021) for “spatial” data management.

**Image analysis in ImageJ (Schindelin *et al.* 2012)**

The whole process is available in a Macro used in Fiji (provided in a separate file).

The images, obtained from our 2-step pre-treatment, are first converted to 8-bit images, then a Kuwahara smoothing filter is used to reduce the noise in the image, which remove bubble artifacts while preserving the edges, i.e., the hyphae. Here we used a Kuwahara linear filter, which is a variant of this adaptive noise reduction filter. Then a Sobel edge detector is used to detect drastic changes in intensity, i.e., the hyphae. Then the “edge-image is converted into a binary mask that makes the fungal filament edge appear white and the background black. Finally, we run a fractal box count algorithm with box sizes of 1, 2, 4, 8, 16, 32, 64, 128, 256, 512 pixels wide. For each grid made of a given box size, each box covering a white pixel is counted. Repeated over the range of box size, the fractal dimension D of the image can be then calculated.

Note for using the Fiji Macro: The input folder contains all the cropped images, whatever the experimental modality. The output folder contains all the related images (saved at line 8 of the macro), on which the fractal box count algorithm is applied. At the end a “results data-sheet” is subsequently saved, storing the results of all images computed from the input folder.

When looking at the images in the output folder, one can see that the boundary resulting from the cropping step is identified as an edge. A test was performed (but not presented here) on 4 samples to define if this artefact influences the calculation of D or not. For such a test, the cropping hand-made polygons defined and saved for each image in R, was imported into imageJ. Then, by a single step of erosion of the polygon-based binary image we obtained, we were able to crop the edge-image (before it was created as a binary mask). The obtained image was free of the cropping artefact and can be compared to the same image with artefact. No fractal dimension difference was detected, so this time-consuming computing task was discarded.

Attached: files:

*Script_2crop_clean.R* first module in R

*png_crop_by_polygon_clean.R* second module in R

*For_FD_with_Fiji.ijm* macro program to run into Fiji (additional file)

References:

Barthelme, S. (2020). imager: Image Processing Library Based on 'CImg'. R package version 0.42.3. https://CRAN.R-project.org/package=imager

Hijmans, R.J. (2021). raster: Geographic Data Analysis and Modeling. R package version 3.4-10. https://CRAN.R-project.org/package=raster

Ooms, J. (2020). magick: Advanced Graphics and Image-Processing in R. R package version 2.3. https://CRAN.R-project.org/package=magick

Pebesma, E.J., R.S. Bivand, 2005. Classes and methods for spatial data in R. R News 5 (2), <https://cran.r-project.org/doc/Rnews/>

R Core Team (2017). R: A language and environment for statistical computing. R Foundation for Statistical Computing, Vienna, Austria. URL <https://www.R-project.org/>

Schindelin, J., Arganda-Carreras I., Frise E., Kaynig V., Longair M., Pietzsch T., Preibisch S., Rueden C., Saalfeld S., Schmid B., Tinevez J-Y, White D.J., Hartenstein V., Eliceiri K., Tomancak P., Cardona, A. (2012). Fiji: an open-source platform for biological-image analysis. Nature Methods, 9(7), 676–682. doi:10.1038/nmeth.2019

*Script_2crop_clean.R* first module in R

rm(list=ls())

library(imager)

library(magick)

library(raster)

library(sp)

# running with R 4.x

setwd("PUT_YOUR_FOLDER_PATH_HERE")

#here we have only tif images

Files <- list.files(pattern="tif$")

paths <- paste(getwd(), "/", Files, sep="")

for(i in 1:length(paths)){

# read with imageMagick

im_IMgck <- image_read(paths[i])%>% image_quantize(colorspace = 'gray')

image_write(im_IMgck, path=gsub(".tif", ".png", paths[i]), format="png")

im <- load.image(gsub(".tif", ".png", paths[i]))

#Manual removal of the droplets

if(unlist(attributes(dev.cur()))!="null device"){sapply(1:length(c(dev.list())), function(i)dev.off(dev.cur()))}

plot(im)

# hand-made polygon for cropping, you can make polygon as complex as you want (no hole supported here)

# add points by pressing the (first) mouse button,

# end the polygon building by pressing any other mouse button (or esc key for some graphic devices).

Manual <- locator( type="l", col=2)

xym <- do.call(cbind, Manual)

p = Polygon(xym)

ps = Polygons(list(p),1)

MASK = SpatialPolygons(list(ps))

# cropping image to obtain a raster

im_IMgck_array <- as.integer(im_IMgck[[1]])

int <- matrix(im_IMgck_array, ncol=dim(im)[1], byrow=F)

data_raw <- raster(int)

extent(data_raw)@xmax <- dim(im)[1]

extent(data_raw)@xmin <- 1

extent(data_raw)@ymax <- dim(im)[2]

extent(data_raw)@ymin <- 1

r <- raster()

proj4string(r) <- CRS(sprintf("+proj=aeqd +lat_0=%s +lon_0=%s +x_0=0 +y_0=0 +R=6371000", as.integer(dim(data_raw)[2])/2, as.integer(dim(data_raw)[1])/2))

#r[is.na(r)] <- 1

r[]<-NA

extent(r)@xmin <- extent(data_raw)@xmin

extent(r)@xmax <- extent(data_raw)@xmax

extent(r)@ymin <- extent(data_raw)@ymin

extent(r)@ymax <- extent(data_raw)@ymax

r@ ncols <- as.integer(dim(data_raw)[2])

r@ nrows <- as.integer(dim(data_raw)[1])

rp <- rasterize(MASK, r)

test <- as.matrix(rp)

fliplr = function(x) {

x[,ncol(x):1]

}

test <- t(test)

test <- fliplr(test)

Mask <- t(raster(test))

extent(Mask)@xmax <- dim(im)[1]

extent(Mask)@xmin <- 1

extent(Mask)@ymax <- dim(im)[2]

extent(Mask)@ymin <- 1

Clean_data <- mask(data_raw, Mask, matrix=NA)

plot(Clean_data)

Sys.sleep(1)

############### end droplet cleaning

# save important data

DAT <- list(MASK, Clean_data, paths[i])

save(DAT, file=gsub(".tif", ".RData", gsub(paste(getwd(), "/", sep=""), "", paths[i])))

}

*png_crop_by_polygon_clean.R* second module in R

library(stringr)

library(imager)

library(magick)

# running with R 4.x

PWD <- "PATH_TO_YOUR_FOLDERS"

setwd(PWD)

# get the folder with the images to analyse (HERE WE HAVE MANY FOLDER IN PATH_TO_YOUR_FOLDERS)

folders <- "MY_FOLDER_OF_CHOICE"

OK <- sapply(1:length(folders), function(i){

setwd(paste(getwd(), folders[i], sep="/"))

#list RDA we created in the first step

LIST_RData <- list.files(pattern="RData")

OK <- sapply(1:length(LIST_RData), function(j){

#open each to crop images or save raster as image format png

load(LIST_RData[j])

#conversion to png for imager

im <- image_read(paste(getwd(), tail(unlist(str_split(DAT[[3]], "\\/")), 1), sep="/"))

tmpF <- tempfile(fileext=".png")

im <- image_convert(im, format="png")

image_write(im, path=tmpF, format="png", quality =100)

#to work in imager

im <- load.image(tmpF)

FOR_mask <- !is.na(as.cimg(DAT[[2]])) %>% pixset

#duplicate chanels for futher use

final <- im* add.colour(FOR_mask)

px <- add.colour(!FOR_mask)

im <- final

#make a uniform image of the same dimensions

#using a background colour of R=1,G=1,B=1 (white)

bg <- imfill(dim=dim(final),val=c(1,1,1))

msk <- as.cimg(px)

final <- bg*msk+(1-msk)*im

save.image(final,file=gsub("tif$", "png", paste(getwd(), tail(unlist(str_split(DAT[[3]], "\\/")), 1), sep="/")))

})

setwd(PWD)

})
